# The central role of creatine and polyamines in fetal growth restriction

**DOI:** 10.1096/fj.202401946R

**Published:** 2024-11-30

**Authors:** Eros Di Giorgio, Serena Xodo, Maria Orsaria, Laura Mariuzzi, Raffaella Picco, Vanessa Tolotto, Ylenia Cortolezzis, Francesca D'Este, Nicole Grandi, Lorenza Driul, Ambrogio Londero, Luigi E. Xodo

**Affiliations:** ^1^ Department of Medicine University of Udine Udine Italy; ^2^ Clinic of Obstetrics and Gynecology Santa Maria della Misericordia Hospital, ASUFC Udine Italy; ^3^ Institute of Pathology, Department of Medicine University of Udine Udine Italy; ^4^ Laboratory of Molecular Virology, Department of Life and Environmental Sciences University of Cagliari Cagliari Italy; ^5^ Obstetrics and Gynecology Unit IRCCS Institute Giannina Gaslini Genova Italy

**Keywords:** creatine metabolism, fetal growth restriction, metabolome, placenta biopsies, placenta organoids, polyamine metabolism, spermidine/spermidine N1‐acetyltransferase 1, transcriptome

## Abstract

Placental insufficiency often correlates with fetal growth restriction (FGR), a condition that has both short‐ and long‐term effects on the health of the newborn. In our study, we analyzed placental tissue from infants with FGR and from infants classified as small for gestational age (SGA) or appropriate for gestational age (AGA), performing comprehensive analyses that included transcriptomics and metabolomics. By examining villus tissue biopsies and 3D trophoblast organoids, we identified significant metabolic changes in placentas associated with FGR. These changes include adaptations to reduced oxygen levels and modifications in arginine metabolism, particularly within the polyamine and creatine phosphate synthesis pathways. Specifically, we found that placentas with FGR utilize arginine to produce phosphocreatine, a crucial energy reservoir for ATP production that is essential for maintaining trophoblast function. In addition, we found polyamine insufficiency in FGR placentas due to increased SAT1 expression. SAT1 facilitates the acetylation and subsequent elimination of spermine and spermidine from trophoblasts, resulting in a deficit of polyamines that cannot be compensated by arginine or polyamine supplementation alone, unless SAT1 expression is suppressed. Our study contributes significantly to the understanding of metabolic adaptations associated with placental dysfunction and provides valuable insights into potential therapeutic opportunities for the future.

## Graphical abstract

Placental insufficiency is often associated with FGR. Placental biopsies and organoids (PlOs) from AGA, SGA, and FGR placentas were analyzed using transcriptomics and metabolomics. Significant metabolic changes were detected in FGR placentas and PlOs, including adaptations to low oxygen levels and altered arginine metabolism in polyamine and creatine phosphate synthesis. FGR placentas utilize arginine to produce phosphocreatine for ATP. Moreover, polyamine deficiency in FGR results from increased SAT1 expression which leads to a decrease in the intracellular bioavailability of spermine and spermidine in the trophoblasts.

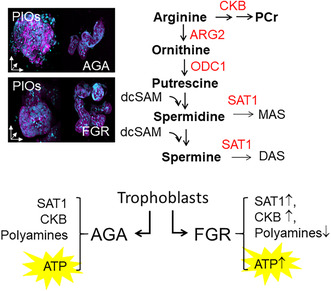

**Abbreviations**
AGA: Appropriate for gestational age
DAF‐2DA: 4,5‐diaminofluorescein‐2‐diacetate
DAMs: Differentially accumulated metabolites
DAS: N1, N8‐diacetylspermidine
DEGs: Differentially expressed genes
DFMO: Difluoromethylornithine
DMEM: Dulbecco Modified Eagle Medium
EVT: Extravillous trophoblasts
FGR: Fetal growth restriction
GAA: Guanidinoacetate
GO: Gene ontology
GSEA: Gene set enrichment analysis
MAS: N1‐acetylspermidine
NO: Nitric oxide
OXPHOS: Oxidative phosphorylation
PCA: Principal component analysis
PE: Preeclampsia
PlOs: Placenta organoids
PPP: Pentose phosphate pathway
RNA‐seq: RNA sequencing
RT‐qPCR: Quantitative reverse transcription polymerase chain reaction
SGA: Small for gestational age
STB: Syncytiotrophoblasts
TB: Trophoblasts
TOM: Trophoblast organoid medium
UC: Urea cycle

## 1 INTRODUCTION

Fetal growth restriction (FGR) refers to inadequate fetal growth compared to the expected individual potential.^1^, ^2^ Clinically, a fetus is classified as small for gestational age (SGA) when its ultrasonographic size is below the 10th percentile for its gestational age.^3^, ^4^ In contrast, a fetus is defined as growth‐restricted when its estimated weight or abdominal circumference is below the third percentile, with additional criteria such as Doppler abnormalities, as outlined in a 2016 Delphi consensus.^5^, ^6^ This distinction is crucial, as fetuses with pathological growth restriction have a significantly increased risk of adverse outcomes, including prematurity, developmental delays, learning disabilities, and adult noncommunicable diseases.^7^, ^8^, ^9^, ^10^, ^11^

Fetal growth restriction (FGR) can result from various factors, with placental dysfunction being the predominant cause, responsible for the majority of cases. Other factors, including genetic disorders, chromosomal abnormalities, and congenital infections, account for approximately 10% of cases.^12^, ^13^ In normal pregnancies, there is considerable remodeling of the maternal spiral arteries in the first trimester to ensure an adequate blood supply to the developing placenta. There is increasing evidence that early‐onset FGR is due to poor placentation and incomplete remodeling of the uteroplacental spiral arteries. This leads to reduced uteroplacental blood flow, resulting in decreased oxygen and nutrient supply to the fetus, causing hypoxia, oxidative stress, and impaired fetal growth.^14^, ^15^ In contrast, late FGR is more likely to be due to acquired placental dysfunction, which occurs when the maternal cardiovascular system is no longer able to respond to the increased demands of the placenta. The prognosis and management of FGR are complex and depend on the underlying pathology, the variety of causes, the severity of ultrasound findings, the gestational age at onset, and the presence of other maternal conditions. Thus, FGR imposes significant societal and economic burdens on individuals and healthcare systems globally.

Despite a robust clinical characterization, the molecular mechanisms leading to FGR remain poorly understood. Previous studies have identified differential gene expression related to angiogenesis,^16^ apoptosis,^16^, ^17^ placental trophoblast secretion and cell adhesion,^18^ hypoxia,^19^ and splicing.^20^ In our study, we combined unbiased next‐generation RNA sequencing with metabolomic analysis to identify metabolic differences between FGR/SGA placentas and those from appropriate gestational age (AGA) newborns. To enhance the characterization of the placental transcriptome, we compared gene expression patterns of our FGR group with those from the Pregnancy Outcome Prediction (POP) study.^21^ Our RNA‐seq and metabolomic data, along with experiments on placental organoids, revealed alterations in the metabolic pathways of creatine and polyamines, along with upregulation of hypoxia‐related metabolic pathways. Understanding the actual effects of creatine and polyamine metabolic pathways in the dysfunctional FGR placenta will be an important research topic for future work.

## 2 MATERIALS AND METHODS

### 2.1 Study participants

Women attending routine ultrasound examinations during pregnancy were recruited for this study. Inclusion criteria were age of 18 years or older, a singleton pregnancy, a live fetus at the time of the ultrasound scan, and proficiency in Italian. Exclusion criteria included the antenatal detection of fetal abnormalities, maternal genetic diseases, uncompensated endocrine or cardiovascular disorders, multiple pregnancies, and severe maternal psychiatric illness or history of drug abuse. Routine first‐ and second‐trimester screenings were performed on all pregnancies. In cases where structural anomalies were detected, invasive prenatal tests—such as amniocentesis or chorionic villus sampling—were conducted. In addition, in cases of early diagnosis of FGR, patients were offered invasive test to perform standard karyotyping and comparative genomic hybridization (CGH) array. As part of the routine FGR management protocol, pregnancies complicated by FGR were also screened for infections, including toxoplasmosis, rubella, cytomegalovirus, and herpes simplex virus, as these infections have the potential to cause placental and/or congenital infections. Cases of FGR not attributable to placental insufficiency were excluded. The fetal growth was monitored using standard reference charts, specifically the Hadlock fetal weight tables.^22^ Moreover, fetal Doppler measures were analyzed according to the reference tables of Arduini and Rizzo^23^ and Bashat and Gembruch.^24^ The estimates of fetal growth just before birth were confirmed by neonatal birth weight using the Italian postnatal growth standards.^25^

Finally, this study was approved by the Hospital Review Board (ASUFC, decree no. 289 of 17/03/2021) and complied with the standards of the Italian Data Protection Authority for scientific research and the Declaration of Helsinki.

### 2.2 Sample collection

All experiments were performed on placental biopsies from male and female newborns as described in the main text. The transcriptomic studies were performed on 23 term placentas, which were categorized into three groups: placentas from newborns with appropriate weight for gestational age (AGA) (*n* = 9); placentas from newborns with birth weight below the 10th percentile for gestational age (SGA) (*n* = 9); and placentas from newborns with an ultrasonographic diagnosis of fetal growth restriction according to ISUOG guidelines (FGR) (*n* = 5). The sample size for RNA‐seq was determined according to RNASeqPS (http://cqs.mc.vanderbilt.edu/shiny/RNAseqPS/), while for metabolomic studies, the minimum sample size required by the MetaboLights database was used. Newborns with FGR not due to placental insufficiency were excluded from the study as all pregnancies underwent routine first trimester screening and, if necessary, genetic testing.

**FIGURE 1:**
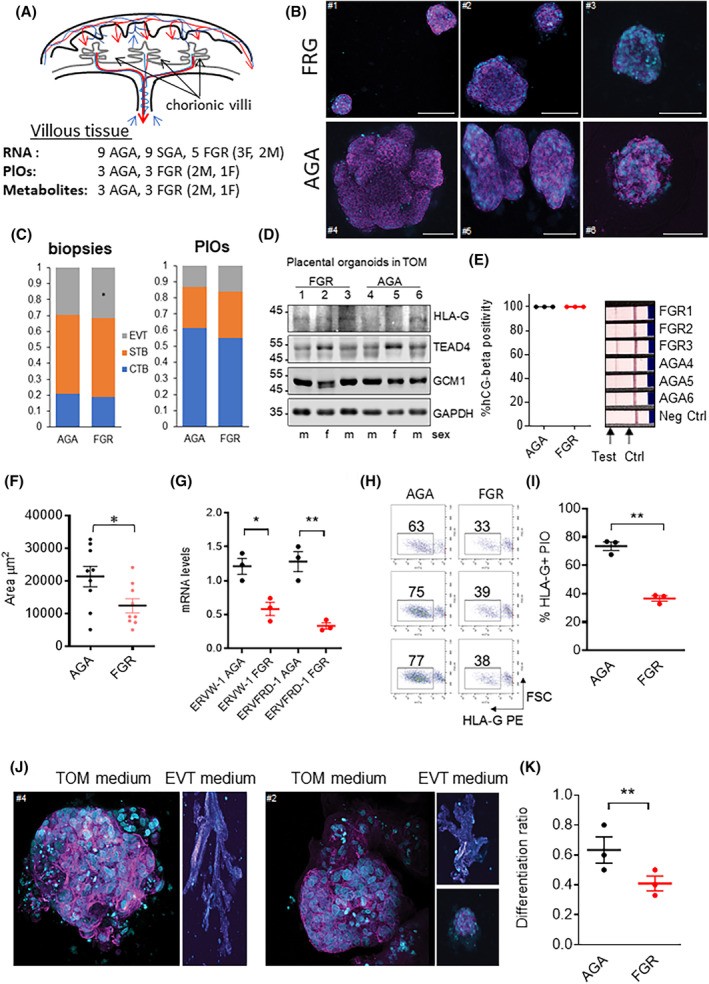
Placental biopsy sampling and characterization of FGR and AGA organoids. (A) Schematic representation of the human placenta, composed of a series of highly branched structures, called villi, which directly bath in maternal blood; scheme of the samples used in this study. (B) Trophoblasts PlOs from FGR and AGA placentas (>32 weeks of gestation) were obtained from villous tissue as reported in Ref. [26] and cultured for 11 day in TOM; Phallodin‐AF546 and Hoechst 33342 were used to stain actin cytoskeleton (magenta) and DNA (cyan). Scale bar 100 μM. (C) Proportions of trophoblast cell type (CTB, STB, and EVT) in placental biopsies and PlOs. (D) Western blot showing the expression of the transcription factors HLA‐G, TEAD4, and GCM1 in AGA and FGR PlOs. GAPDH was used as loading control. (E) Positivity to hCG beta pregnancy test; pregnancy test strips were immersed in PlO culture medium for a few seconds. HEK293 cell culture medium was used as a negative control. (F) Scatter plot representing the area (μm^2^) of AGA and FGR PlOs reached after 11 days of culture in TOM; mean and SD are indicated, *n* = 9. (G) mRNA relative levels of ERVW‐1 and ERVFRD‐1 determined by RT‐qPCR in the AGA and FGR PlOs. Mean and standard deviation are indicated. (H, I) Representative cytofluorimetric dot plot of HLA‐G PE in AGA and FGR PlOs and scatter plot representing HLA‐G positivity in AGA and FGR PlOs at the end of the differentiation protocol (11 days in EVT medium +7 7 days without NRG‐1). Means and SD are reported. (J) Representative 3D reconstruction images obtained by confocal imaging obtained from AGA and FGR PlOs maintained in TOM or EVT medium. (K) Dot plot representing the differentiation rate of AGA and FGR PlOs. For each biological sample, the mean value of the rate of matrix invading organoids (FGR samples: 1, 2, 3 and AGA samples: 4, 5, 6) was plotted. Mean and SD are indicated. All data reported represent the mean ± SD of at least 3 independent experiments: *p ≤ .05; **p ≤ .01; and ***p ≤ .001 by Student's *t*‐test between the indicated pairwise comparisons.

Villous tissue was isolated, subjected to thorough washing of maternal blood with saline solution, and promptly frozen at −80°C until further use. Additionally, 3D organoids were derived from the villous tissue of three AGA placentas and three FGR placentas, while 50% of the remaining biopsies was subjected to metabolomic studies (Figure 1A).

### 2.3 Establishment, maintenance, and differentiation of placental organoid culture

Trophoblast organoids from AGA and FGR placentas obtained by late gestations (>32 weeks of gestation) were established starting from villous tissue biopsies following the protocol by Turco lab, with some modifications.^26^ Briefly, villi were scraped from the chorionic membrane with a scalpel and collected with Advanced DMEM/F‐12 (Thermo Fisher). The cell suspension was collected by centrifugation (1300 rpm, 5′), disaggregated with 0.5 mL of 0.2% trypsin‐250/0.02% EDTA (37°C, 5′), quenched with 1.5 mL Advanced DMEM/F‐12, filtered through a 100‐μm cell strainer (Thermo Fisher), pelleted by centrifugation (1300 rpm, 5′), then resuspended with 10× volume of ice‐cold reduced growth factors Cultrex (Trevigen), and plated in 24‐well plates (4 × 13μL matrix drops per well). Each drop was supplemented with 250 μL of trophoblast organoid medium (TOM) (Advanced DMEM/F12, 1× N2 supplement, 1× B27 supplement, Primocin 100 μg/mL, N‐acetyl‐L‐cysteine 1.25 mM, L‐glutamine 2 mM, recombinant human EGF 50 ng/mL, CHIR99021 1.5 μM, recombinant human R‐spondin‐1 80 ng/mL, recombinant human HGF 50 ng/mL, A83‐01500 nM, prostaglandin E2 2.5 μM, Y‐27632 2 μM). PlOs were cultured for several weeks and passaged every 7–10 days. To achieve EVT differentiation, PlOs were incubated for 11 days in EVT medium (Advanced DMEM/F12, L‐glutamine 2 mM, 2‐mercaptoethanol 0.1 mM, penicillin/streptomycin solution 0.5% (vol./vol.), BSA 0.3% (vol./vol.), ITS‐X supplement 1% (vol./vol.), NRG1 100 ng/mL, A83‐01 7.5 μM, knockout serum replacement 4% (vol./vol.)) and during the following 7 days in EVT medium without NRG1. Trophoblast subpopulations in the placenta biopsies and PlOs were identified by measuring the expression of specific biomarkers using qPCR: TP63, XRCC6, and SRSF2 for cytotrophoblasts (CTB); CGA, PSG2, and HSD3B1 for syncytiotrophoblasts (STB); and MMP2, ITGA5, and FSTL3 for extravillous trophoblasts (EVT).^27^ The absolute expression levels (TPM) of the individual markers were determined using RNA‐seq data. The average expression level of the three markers representing the three subpopulations was calculated and used to determine the proportionality of representation of each subpopulation by applying the following formula: CTB = CTB/(CTB + STB + EVT)*100; STB = STB/(CTB + STB + EVT)*100; EVT = EVT/(CTB + STB + EVT)*100. The comparative CT method was applied to compare the expression levels of each marker between each organoid and determine their representativeness with respect to the subpopulations.

Arginine deprivation metabolic studies were carried out using SILAC DMEM Flex Media (Gibco, MA USA) in place of Advanced DMEM/F‐12. PlOs were maintained at 37°C, 5% CO_2_, and 21% O_2_. When indicated, PlOs were incubated at 2% O_2_ or 8% O_2_ for the indicated time in InViVo_2_ Ruskinn hypoxic chamber (Ruskinn, UK). The following chemicals were used: DFMO (CAS n. 96020‐91‐6, Merck, Germany), Cyclocreatine (35404‐50‐3, Cayman, USA), Y‐27632 (CAS n. 129830‐38‐2, Merck, Germany), CHIR99021 (CAS n. 252917‐06‐9, Merck, Germany), and A83‐01 (CAS n. 909910‐43‐6, Merck, Germany). Cytokines and growth factors were from PeproTech (UK).

### 2.4 Lentiviral infection of PlOs

HEK‐293T cells were transfected with 20 μg pLKO plasmids expressing shCT, shSAT1 A (TRCN0000035250, Sigma‐Aldrich), shSAT1 B (TRCN0000035252, Sigma‐Aldrich), 10 μg psPAX2 (Addgene plasmid #12260), 3.6 μg pMD2.G (Addgene plasmid #12259), and 100 μL PEI (1 mg/mL, MW 25000 Da, Merck). After 36 h and 72 h, the medium was collected, filtered through a 0.45‐μm PES filter, pooled, and added with polybrene (8 μg/mL). Then, viral particles were applied to a single‐cell suspension obtained by enzymatic dissociation of AGA and FGR PlOs. After 8 h of infection, cells were pelleted, embedded in Cultrex, and plated. In the absence of a well‐functioning and specific antibody against SAT1, the efficacy of silencing was tested by qRT‐PCR.

### 2.5 Cytofluorimetric analysis

Nitric oxide levels in PlOs were quantified as follows: AGA and FGR PlOs cultured in 12‐well plates were washed twice with PBS and then incubated for 30 min with 3 μM DAF‐FM DA (Invitrogen, USA) in phenol red‐free Advanced DMEM/F‐12 without serum. After two additional PBS washes, the cells were harvested and resuspended in 200 μL PBS, and single‐cell suspensions were analyzed using a BD FACSCalibur flow cytometer (488 nm argon laser, FL1 channel).

For HLA‐G positivity detection, PlOs were cultured in EVT medium for 11 days and in EVT medium lacking NRG‐1 for 7 days. Single‐cell suspensions were then collected, incubated for 30 min in 100 μL PBS containing 3 μL monoclonal anti‐HLA‐G antibody (87G)‐PE (eBioscience, Invitrogen, USA), and analyzed using a BD FACSCalibur flow cytometer (488 nm argon laser, FL2 channel).

### 2.6 Immunofluorescence and immunoblotting

Placental organoids were fixed with 3% paraformaldehyde and permeabilized with 0.3% Triton X‐100. Actin was labeled with phalloidin‐AF546 (Molecular Probes, USA). PlOs were imaged with a confocal microscope Leica TCS SP8X. Nuclei were stained with Hoechst 33342 (10 μg/mL, Merck). Images represent maximum intensity projections of 3D image stacks and were adjusted for brightness and contrast for optimal visualization. For the preparation of protein lysates, placental biopsies were frozen in liquid nitrogen, ground to a powder with a pestle, and lysed for 1 h at 4°C with 200 μL RIPA lysis buffer for 1 mg tissue. 4× Laemmli sample buffer was added to the clarified lysates, and after boiling, the samples were loaded onto SDS/PAGE gels. The cell lysates were incubated with primary antibodies after SDS–PAGE and immunoblotting on nitrocellulose (Whatman). The following primary antibodies were used: anti‐TEAD4 (HPA056896, Merck), anti‐GCM1 (HPA011343, Merck), anti‐HLA‐G (E8N9C, Cell Signaling), and anti‐GAPDH (97166, Cell Signaling). HPR‐conjugated secondary antibodies were purchased from Cell Signaling, and the blots were developed using Super Signal West Dura (Thermo Fisher Scientific).

### 2.7 Metabolite extraction and liquid chromatography–mass spectrometry of label‐free metabolites

Placental villous tissue from AGA and FGR groups was harvested, flash frozen, and stored at −80°C. Samples were thawed on ice, homogenized in a ball‐mill grinder (30 Hz for 30 s), and mixed with 400 μL of methanol:water (7:3) solution containing an internal standard. After shaking at 2500 rpm for 5 min, the mixture was placed on ice for 15 min and then centrifuged at 12000 rpm for 10 min (4°C). 300 μL of the supernatant was stored at −20°C for 30 min, followed by further centrifugation (12000 rpm, 3 min, 4°C). A 200 μL aliquot of supernatant was analyzed using Ultra Performance Liquid Chromatography (UPLC) and Quadrupole‐Time of Flight (TripleTOF 6600+, AB SCIEX). LIT and triple quadrupole (QQQ) scans were acquired on a triple quadrupole–linear ion trap mass spectrometer (QTRAP), QTRAP LC–MS/MS System, equipped with an ESI Turbo Ion‐Spray interface, operating in positive and negative ion mode and controlled by Analyst 1.6.3 software (SCIEX). The ESI source operation parameters were as follows: source temperature 500°C; ion spray voltage (IS) 5500 V (positive), −4500 V (negative); ion source gas I (GSI), gas II (GSII), and curtain gas (CUR) were set at 50, 50, and 25.0 psi, respectively; and the collision gas (CAD) was high. Instrument tuning and mass calibration were performed with 10 and 100 μmol/L polypropylene glycol solutions in QQQ and LIT modes, respectively. Metabolites were quantified by triple quadrupole mass spectrometry with multiple reaction monitoring (MRM). LC/MS run was performed at Metware Biotechnology (Woburn, MA, USA). Analyst 1.6.3 was used to process mass spectrum data. Quantitative analysis was performed with MetwareBio software starting from RAW files. The missing values were first filled in using 1/5 of the minimum value of each row (metabolite), and then, the CV value of the QC sample was calculated, and the metabolites with a CV value less than 0.3 were retained. Bidimensional PCA was conducted to assess variation between groups, and differentially abundant metabolites were identified using VIP (variable importance in projection from OPLS‐DA modeling) > 1 and *p*‐value < .05 (Wilcoxon rank‐sum).

### 2.8 Polyamine and arginine quantification

For polyamine quantification, PlOs were collected in 1 mL of cold PBS by mechanical harvesting. After centrifugation, trophoblasts were collected with 0.1 mL of ice‐cold Polyamine Assay Buffer and lysed for sonication. After incubation for 30′ at 37°C with 0.002 mL of fluorescent polyamine probe (MAK349, ex/em 532/587) (Merck, Italy), fluorescence was detected with Synergy H1 (Biotek, US). Polyamine quantification was determined from comparison with standard curve. The levels of arginine from primary biopsies and PlOs were quantified with L‐Arginine Assay Kit (Merck, MAK370) as previously described.^28^ Medium deprived of exogenous arginine was used for calibration curve.

### 2.9 Measurement of creatine kinase activity and ATP levels

The colorimetric creatine kinase activity assay kit (ab155901, Abcam) and ATP assay kit (Merck, 119107) were used to quantify CK activity and ATP from villus tissue biopsies or PlOs according to the manufacturer's recommendations. Data were normalized to the total protein levels quantified with the Bradford assay.

### 2.10 RNA extraction, quantitative qRT‐PCR, library preparation, and RNA sequencing

Placental biopsies and trophoblast organoids were lysed using TRIzol (Invitrogen, USA). 1.0 μg of total RNA was DNAse I‐treated (Ambion, USA) and retro‐transcribed by using 100 units of M‐MLV Reverse Transcriptase (Life Technologies, USA) in the presence of 1.6 μM oligo(dT) and 4 μM random hexamers (Euroclone, Milan, Italy). qRT‐PCRs were performed using SYBR green technology (KAPA Biosystems). Data were analyzed by comparative threshold cycle (delta delta Ct ΔΔCt) using *HPRT* and *GAPDH* as normalizer. The sequence of primers used for qPCR is reported in Table S1.

For RNA‐seq, 50 ng total RNA was DNAse I‐treated, freed from rRNA (using the ribo‐zero method), and reverse‐transcribed to obtain cDNA. Adapters were ligated to the fragmented cDNA after end repair and A‐tailing according to the DNBSEQ protocol (MGI‐Tech). 16 rounds of PCR amplification were performed to enrich the cDNA fragments. The PCR products were then purified using Ampure XP beads (Agencourt). ssCirDNA was obtained after denaturation and circularization. DNA spheres were generated from each library according to BGI specifications and subjected to paired‐end 100 sequencing at the BGI Genomics facility (BGI Genomics, China).

### 2.11 RNA‐seq analysis and gene set enrichment analysis (GSEA)

Quality control for raw sequencing reads was performed with FastQC (v0.11.9) and MultiQC (v1.09). Alignment of reads was conducted with STAR (v2.7.3a), using the human genome assembly GRCh38 with reference annotation downloaded from Ensembl (version 107). Transcript assembly and quantification were done with StringTie (v2.1.5). A Python script (prepDE.py) was used to extract all the read counts information directly from the files generated in the last step. Differential expression (DE) analysis was performed using gene raw counts, within the R/Bioconductor DESeq2 package. Principal component analysis was carried out with the plotPCA function from the DESeq2 package (v1.28.1). Genes with raw counts mean < 10 were removed from the analysis. Differential expression analysis was performed using DESeq2 with Wald test for significance. Genes with an absolute fold change >1 were considered as differentially expressed. Functional annotation was performed as previously described,^28^ by using ClusterProfiler (v3.16.1) and Enrichr.^29^ Gene set enrichment analysis (GSEA) and the MSigDB database were interrogated to identify significant gene associations. Heatmaps were generated for each gene set, and the expression of each gene was expressed as log2 (fold change) of SGA vs. AGA samples. Leading edge analysis was performed to identify the core gene signature representative of each state. This signature was then launched for functional enrichment analysis by interrogating KEGG and MSigDB Hallmark databases (http://bioinformatics.sdstate.edu/go/).

### 2.12 Statistics

For experimental data, Student's *t*‐test was employed. Mann–Whitney test was applied when normality could not be assumed. *p <* .05 was chosen as statistical limit of significance. For comparisons between more than two samples, ANOVA test was applied coupled to Kruskal–Wallis and Dunn's multiple comparison test. For correlation between two groups, Pearson correlation, Spearman correlation, or Wilcoxon signed‐rank test was calculated for normal or non‐normal distributions, respectively. Excel and GraphPad Prism were used for routineer analysis and R/Bioconductor packages for large data analysis and heatmap generation. We marked with *p < .05, **p < .01, and ***p < .001. Unless otherwise indicated, all the data in the figures were represented as arithmetic means ± the standard deviations from at least three independent experiments.

## 3 RESULTS

### 3.1 FGR, SGA, and AGA placentas: biopsies and organoids

To investigate the transcriptional and metabolic changes associated with FGR, we used placental biopsies from newborns categorized as FGR, SGA, and AGA based on the last ultrasonographic biometric assessment in the third trimester of pregnancy, between 35 and 37 gestational weeks. The distinction between FGR and SGA fetuses was made in accordance with ISUOG guidelines for the diagnosis and management of SGA fetuses and fetuses with FGR.^3^ RNA‐seq on total RNA from the villous tissue of 9 AGA, 9 SGA, and 5 FGR placentas provided an insight into the transcriptome of both AGA and FGR conditions. Additionally, an unbiased label‐free metabolomic assessment was performed on 3 AGA and 3 FGR biopsies, which were also utilized for the generation of 3D placental organoids (PlOs) (Figure 1A). The clinical characteristics of the cohort of patients for which RNA‐seq data were obtained are reported in Table S2A–C; those of the placentas used for the generation of organoids and for metabolomics are reported in Table S2D. The generation of trophoblast PlOs after >34 weeks of gestation exhibited a comparable success rate of approximately 75% with both AGA and FGR groups (Figure 1B), consistent with prior studies.^28^ Trophoblast subpopulations within the placental biopsies and PlOs were identified by measuring the expression of specific biomarkers using qPCR: TP63, XRCC6, and SRSF2 for cytotrophoblasts (CTB); CGA, PSG2, and HSD3B1 for syncytiotrophoblasts (STB); and MMP2, ITGA5, and FSTL3 for extravillous trophoblasts (EVT)^27^ (Figure S1). These biomarkers were previously characterized through single‐cell RNA sequencing of human blastocysts differentiating into CTB, STB, and EVT,^30^ as well as through single‐cell RNA sequencing of 70 000 cells from first‐trimester placental tissue.^31^ Based on the expression levels of these biomarkers, we estimated the proportions of trophoblast subpopulations in both the biopsies and PlOs (Figure 1C). The biopsies from FGR placenta showed a slight decrease in CTB and a slight increase in EVT compared to the AGA biopsies. The PlOs cultured in Matrigel with TOM showed a strong expansion of the CTB population, as previously described.^26^ This expansion was slightly, but not significantly, reduced in FGR PlOs compared to AGA PlOs. Using Western blot, we also analyzed the levels of TEAD4, GCM1, and HLA‐G—transcription factors specific for CTB, STB, and EVT, respectively—in PlOs cultured in TOM^32^ (Figure 1D). The results showed minimal differences in the expression of these transcription factors between AGA and FGR organoids. Notably, HLA‐G was only weakly expressed under the experimental conditions used, while TEAD4 showed higher expression in PlOs derived from female placentas. All 6 PlOs successfully secreted human chorionic gonadotropin β (hCG‐β) (Figure 1E). Upon cultivation in TOM,^33^ FGR PlOs demonstrated a mean area approximately 40% smaller than that of AGA PlOs (Figure 1F). Furthermore, FGR PlOs exhibited lower mRNA levels of syncytin‐1 (ERVW‐1 gene) and syncytin‐2 (ERVFRD‐1 gene) compared to AGA PlOs (Figure 1G). Previous studies have shown that the application of an extravillous trophoblast (EVT) differentiation protocol enriches the population of HLA‐G+ cells in the PlOs.^26^, ^29^, ^33^ Notably, unlike AGA PlOs, transitioning from TOM maintenance medium to EVT differentiation medium resulted in FGR PlOs with lower enrichment of HLA‐G+ cells (Figure 1H,I) and a reduced proportion of EVT cells that migrated from the PlOs and adhered to the plastic surface (Figure 1J,K). In summary, our findings suggest a deficiency in the self‐renewal and differentiation capacities of FGR placental organoids compared to their AGA counterparts.

### 3.2 RNA sequencing reveals transcriptome differences between FGR and AGA placentas

**FIGURE 2:**
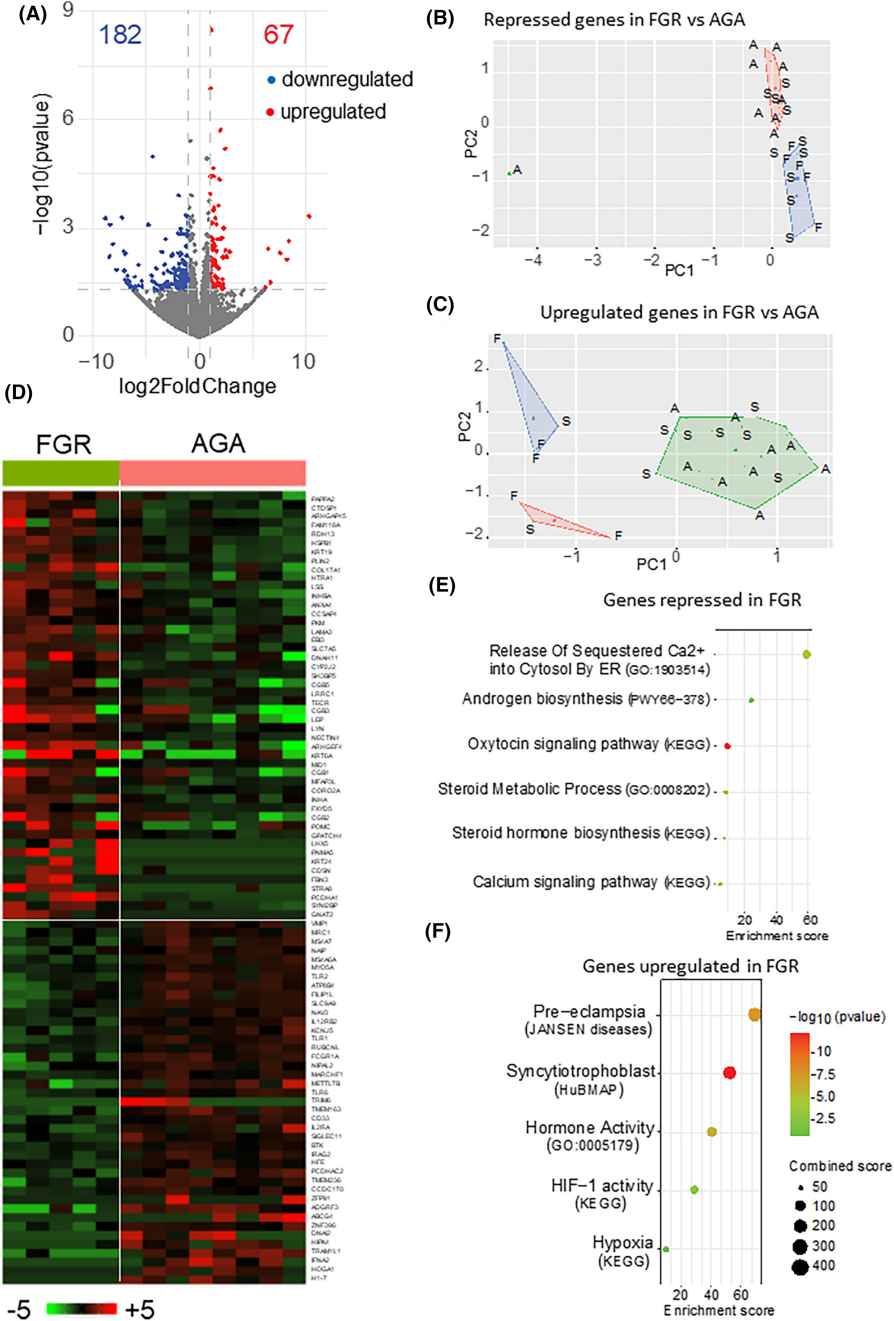
RNA‐seq reveals transcriptomic differences between FGR and AGA placentas. (A) Volcano plot of DEGs in FGR compared to AGA placentas (|log2(Fc)| > 1, *p* < .05). (B, C) PCA of the indicated samples based on the 182 coding genes found to be repressed (B) or the 67 coding genes found to be upregulated (C) in FGR compared to AGA placentas. F = FGR, S = SGA, and A = AGA. (D) Heatmap of the DEGs contributing to more than 50% of difference between FGR and AGA. (E, F) Gene ontology analysis (ShinyGO 0.80) of downregulated and upregulated DEGs identifies enriched terms in FGR. The color and size of the dots are proportional to the significance and strength of the enrichment, as indicated.

Total RNA was extracted from the villous tissue of AGA, SGA, and FGR placentas and subjected to RNA‐seq analysis. Initially, we compared the transcriptomes of the most extreme groups, AGA and FGR, focusing on coding genes. In the FGR group, 249 differentially expressed coding genes (DEGs) were identified compared to the AGA group. The volcano plot shows that among these DEGs, 182 are downregulated and 67 are upregulated, based on a threshold of |log_2_ fold change| ≥ 1 and *p* < .05 (Figure 2A).

Principal component analysis (PCA) shows the separation of FGR samples from AGA samples based on differential expression of genes identified as significantly repressed (Figure 2B) or induced (Figure 2C) in FGR versus AGA. The SGA condition was between the two extremes, as highlighted in the heatmap and hierarchical clustering in Figure S2A,B. The heatmap of DEGs contributing to more than 50% of the difference between FGR and AGA samples is shown in Figure 2D. It shows a net distinction in terms of gene expression between FGR and AGA.

Gene ontology (GO) analysis of the downregulated DEGs revealed six enriched terms, including steroid metabolic process (*p* < 10^−5^), steroid hormone biosynthesis (*p* < 10^−2^), androgen biosynthesis (*p* < 10^−3^), and calcium signaling (*p* < 10^−4^) (Figure 2E), suggesting altered steroidogenesis in FGR placentas.^34^ GO analysis of the upregulated DEGs in FGR placentas identified five enriched terms: preeclampsia (PE) (*p* < 10^−6^), syncytiotrophoblast (*p* < 10^−9^), hormone activity (*p* < 10^−6^), HIF‐1 activity in hypoxia (*p* < 10^−3^), and hypoxia (*p* < 10^−9^) (Figure 2F), indicating hypoxia‐related signaling pathway perturbations in FGR.^35^, ^36^, ^37^

Inhomogeneity characterizes placental samples.^38^, ^39^ To validate our RNA‐seq results, we applied a rigorous bootstrapping method, performing 10 000 permutations of our sample groups using an established bioinformatics pipeline.^39^ This approach identified 4006 DEGs between AGA and FGR groups (Table S3). Functional enrichment analysis after bootstrapping highlighted hypoxia and trophoblast dysfunction in upregulated genes and growth restriction and embryogenetic anomalies in repressed genes (Table S4). Bootstrapping successfully segregated AGA and FGR samples (Figure S2C). In summary, our comprehensive transcriptome analysis, using both conventional and bootstrapping methods, provides compelling evidence for placental dysfunction in FGR.

### 3.3 Identification of gene signatures associated with placental dysfunction in fetal growth restriction

**FIGURE 3:**
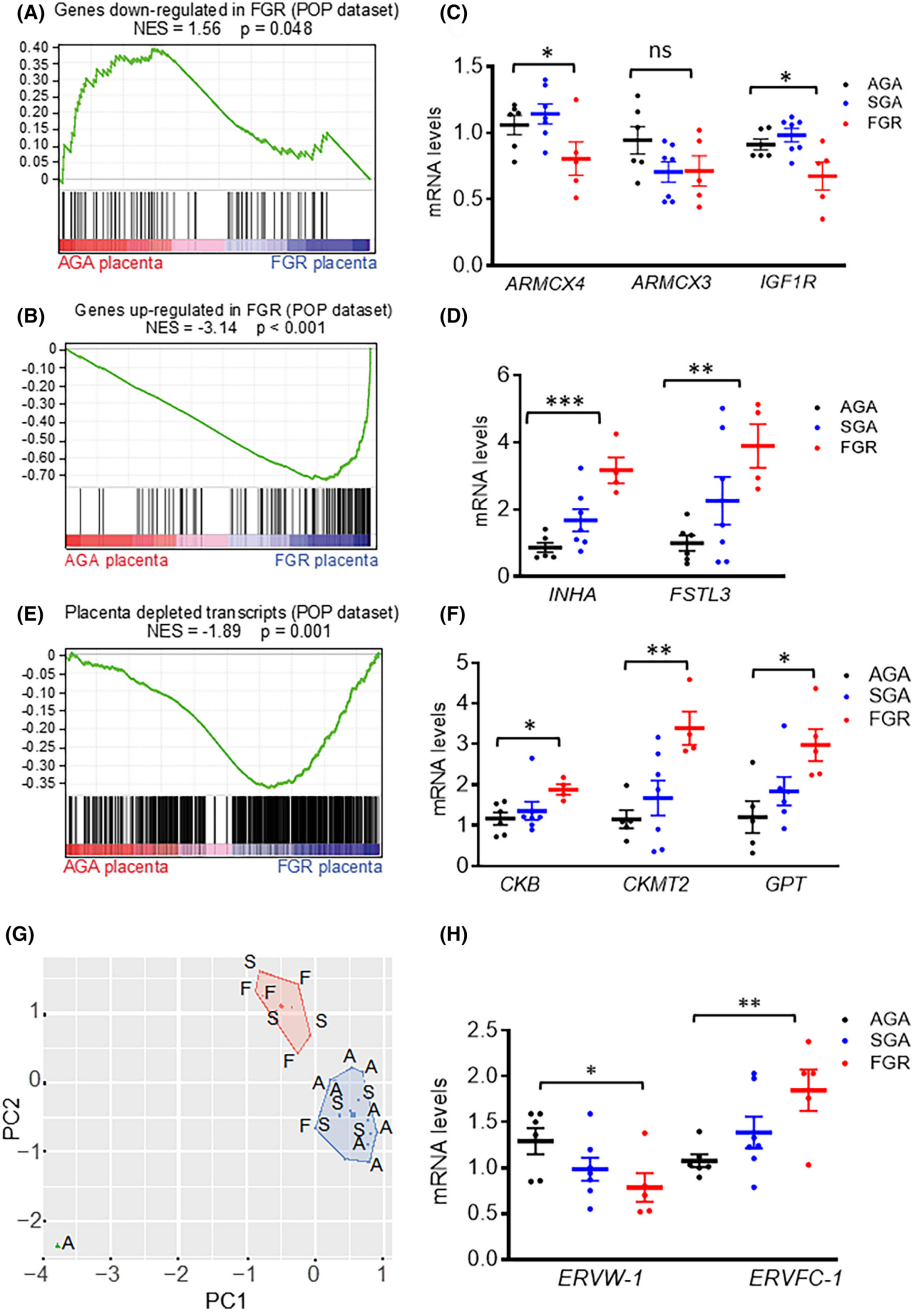
Identification of gene signatures defining FGR. Gene set enrichment analysis (GSEA) performed by using as geneset genes found to be repressed in FGR in POP dataset (ref. 21) and as dataset our RNA‐seq; significant positive enrichment was obtained in AGA vs. FGR comparison. (A,B) GSEA performed by using as geneset genes found to be down‐regulated and upregulated in FGR in POP dataset (ref. 21) and as dataset our RNA‐seq; significant positive enrichment was obtained in FGR vs. AGA comparison. (C, D, F) Scatter plot representing the RT‐qPCR quantification of the indicated mRNA of genes (representative of the functional categories described respectively in A, B, E) between AGA, SGA, and FGR samples; mean, SD, and *p*‐values are indicated. (E) GSEA performed by using as geneset genes found to be depleted in normal placentas (ref. 38) and as dataset our RNA‐seq; significant positive enrichment was obtained in FGR vs. AGA comparison. (G) PCA of the indicated samples, based on the 762 genes found to be depleted in normal placentas with respect to other tissues (ref. 38). F = FGR, S = SGA, and A = AGA. (H) Scatter plot representing the RT‐qPCR quantification of the indicated genes of endogenous retroviral origin; mean and SD are indicated. Data represent the mean ± SD of at least 3 independent experiments: *p ≤ .05; **p ≤ .01; and ***p ≤ .001 by Student's *t*‐test.

To identify core gene signatures commonly altered in the FGR placenta, we conducted a comparative gene expression analysis. Using gene set enrichment analysis (GSEA), we compared the transcriptomes of our FGR placentas with those from the Pregnancy Outcome Prediction (POP) study.^21^ This comparison revealed a convergence of upregulated and downregulated gene sets in both placenta groups (Figure 3A,B). We identified a distinct signature of 78 genes in FGR vs. AGA (38 downregulated and 40 upregulated), contributing to the core enrichment of GSEA, which represents a common FGR signature (Table S5). This signature effectively clusters our FGR placentas (Figures S3A and S4A).

Using the common 78‐gene FGR signature, we performed enrichment analyses against 226 gene‐set libraries using Enrichr.^28^ We identified shared enrichment categories among downregulated genes in both FGR groups (Figure S3B,C), including (i) integral components of plasma membranes (*p* = 8.56 × 10^−4^); (ii) placental development (*p* = 2.08 × 10^−4^); (iii) transmembrane transport (*p* = 1.15 × 10^−3^); and (iv) angiogenesis (*p* = 2.01 × 10^−3^). These findings highlight potential defects in the syncytiotrophoblast basal plasma membrane and altered perfusion in the FGR placenta which can lead to inefficient supply of metabolites.^40^ We verified the diminished expression of three genes (*IGF1R*, *ARMCX3*, and *ARMCX4*) among the 38 commonly repressed genes in the FGR condition. These genes significantly contribute to the functional enrichment of the above categories through a leading edge analysis,^41^ and their downregulation was confirmed by RT‐qPCR (Figure 3C). The *ARMCX* cluster, unique to placental mammals, modulates functions relevant to placentation.^42^

GSEA of the 40 upregulated genes in FGR placentas revealed common enrichment categories linked to (i) preeclampsia (*p* = 2.83 × 10^−10^); (ii) HIF‐1 transcriptional activity in hypoxia (*p* = 1.77 × 10^−7^); and (iii) hypoxia (*p* = 4.54 × 10^−4^) (Figure S4B,C). RT‐qPCR confirmed the upregulation of genes such as inhibin alpha (*INHA*) and follistatin‐like 3 (*FSTL3*) in our FGR placentas (Figure 3D).

Gong et al. reported a significant depletion of transcripts (762 genes) in the human‐term placenta compared to other tissues, highlighting the specialized nature of placental terminal differentiation characterized by the switch‐off of transcripts prevalent in other tissues.^38^ Many of these transcripts govern mitochondrial functions and polyamines metabolism.^38^ GSEA using this set of 762 transcripts showed that our FGR placentas expressed higher levels of these transcripts compared to AGA placentas, suggesting a lack of specialization in FGR placentas (Table S6) (Figure 3E). Functional enrichment analysis revealed categories such as (i) creatine metabolism (*p* = 2.16 × 10^−4^); (ii) creatine kinase complex (*p* = 9.7 × 10^−5^); (iii) arginine and proline metabolism (*p* = .115); and (iv) mitochondrial ribosome (*p* = 1.1 × 10^−4^), which were shared by depleted genes in the healthy placentas from the POP dataset and upregulated genes in our FGR group (Table S5). This important finding suggests that genes involved in the creatine and polyamine pathways, typically silenced in healthy placentas, are instead active in FGR placentas. RT‐qPCR confirmed the upregulation of genes involved in creatine metabolism, such as creatine kinase (*CKB*), creatine kinase mitochondrial 2 (*CKMT2*), and glutamate pyruvate transaminase (*GPT*) in FGR placentas (Figure 3F). PCA and heatmap of the genes depleted in the healthy placentas from the POP dataset and upregulated in our FGR group showed clear clustering, with AGA placentas separate from FGR placentas and SGA placentas in between (Figures 3G and S5). Moreover, the altered expression of *ERVW*‐1 and *ERVFC*‐1, two genes of retroviral origin involved in placenta maturation,^40^, ^41^, ^42^ supports the idea that the FGR placenta is less mature compared to AGA placenta (Figure 3H). Overall, our transcriptome analysis of FGR placentas showed excellent overlap with the FGR placentas from the public POP dataset, highlighting the activation of hypoxia and alteration of arginine metabolism in both groups.

### 3.4 Metabolomic insights in FGR placenta confirmed hypoxia and reprogramming in arginine metabolism

An unbiased metabolomic analysis performed on three FGR and three AGA placental biopsies allowed us to quantify 1165 metabolites and provided valuable insights into the metabolic changes associated with FGR. PCA showed a clear separation between the AGA and FGR groups (Figure S6). Notably, 19.4% of the quantifiable metabolites exhibited differential abundance between the two groups. Specifically, 152 differentially accumulated metabolites (DAMs) were found in lower abundance and 74 in higher abundance in FGR placentas compared to AGA placentas. GO and KEGG enrichment analyses revealed that the upregulated metabolites in FGR were enriched in nicotinate and nicotinamide metabolism (*p* = 7.24 × 10^−4^), glycerophospholipid metabolism (*p* = 9.5 × 10^−3^), and arginine and proline metabolism (*p* = 1.4 × 10^−2^). In contrast, the downregulated DAMs were enriched in steroid biosynthesis (*p* = 2.79 × 10^−4^), taurine and hypotaurine metabolism (*p* = 1.10 × 10^−2^), and glutathione metabolism (*p* = 3.2 × 10^−2^) (Table S7). These metabolomic findings align consistently with the RNA‐seq data depicted in Figure 2. Both transcriptome and metabolome analyses of FGR placentas underscored metabolic adaptations to hypoxia, alterations in arginine metabolism, and a reduction in placental steroid hormone synthesis.

### 3.5 FGR placentas activate a transcriptional short circuit that supports metabolic adaptation to hypoxia

**FIGURE 4:**
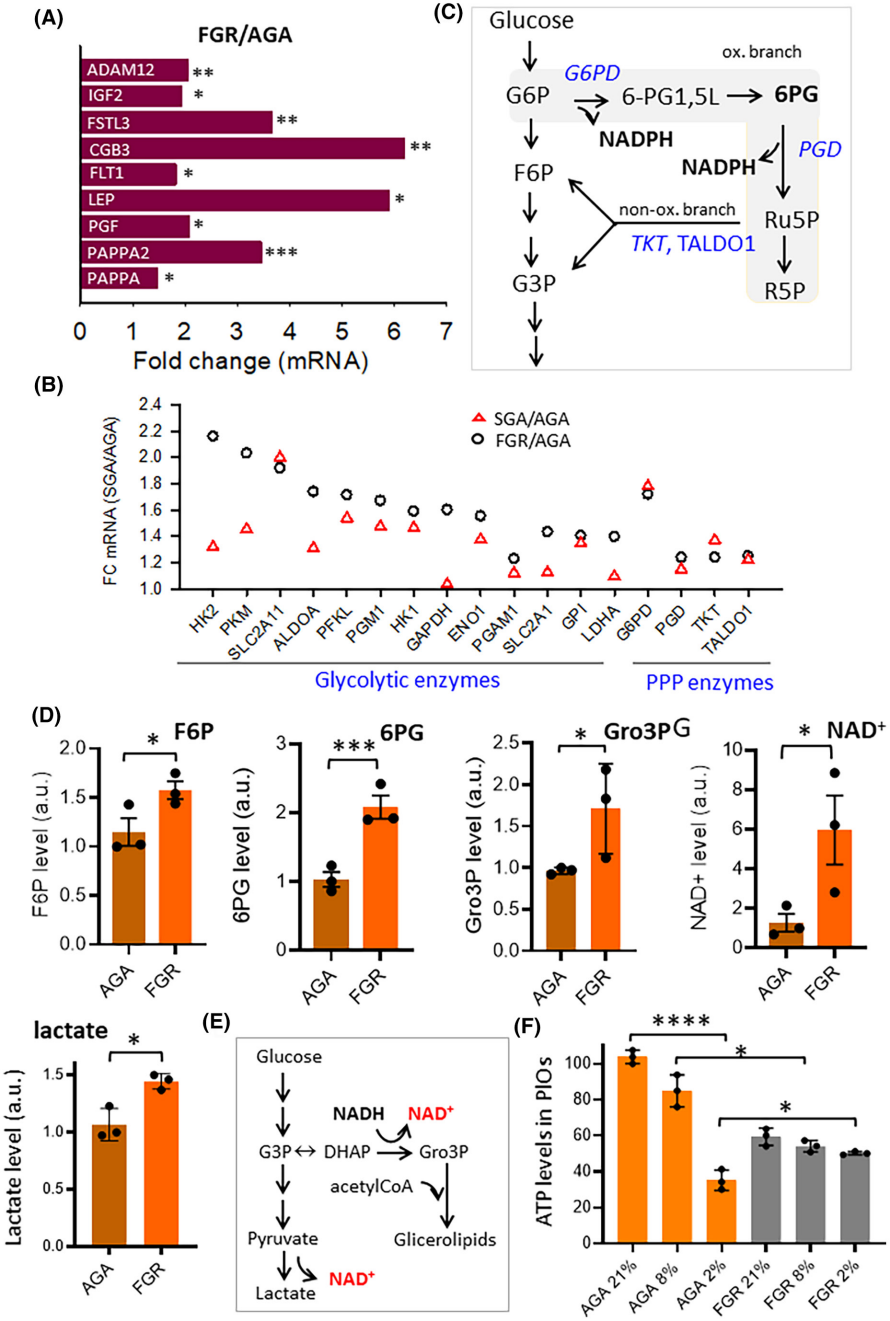
Hypoxia triggers metabolic reprogramming in FGR. (A) Expression level of specific hypoxia‐related genes determined by RNA‐seq analysis. The fold change of FGR mRNA/ AGA mRNA is reported. (B) Fold change of the indicated genes involved in glycolysis and PPP pathways. Red triangles indicate the fold change obtained from SGA vs. AGA comparison; black circles indicate the fold change obtained from FGR vs. AGA comparison. (C) Schematic representation of the integration of glycolysis and PPP; genes encoding for key rate‐limiting enzymes found to be de‐regulated in FGR are highlighted in blue. (D) Relative levels of the indicated metabolites determined by mass spectrometry in AGA and FGR placentas. F6P: D‐Fructose 6‐phosphate; 6PG: 6‐Phosphogluconic acid; Gro3P: Glycerol‐3‐phosphate; and NAD^+^: Oxidized Nicotinamide Adenine Dinucleotide. (E) Gro3P pathway and LDHA can regenerate NAD^+^ for maintaining glycolysis and PPP. (F) Levels of ATP in AGA and FGR organoids cultured for 72 h under different oxygen concentrations: 2, 8, and 21 O_2_%, as indicated. Data represent the mean ± SD of at least 3 independent experiments: *p ≤ .05; **p ≤ .01; and ***p ≤ .001 by Student's *t*‐test.

In early pregnancy, particularly during the first trimester, the placenta develops under hypoxic conditions, essential for the differentiation of extravillous trophoblasts (EVT) until complete vascularization occurs by the trimester's end.^43^, ^44^ However, complications like FGR and preeclampsia (PE) can maintain hypoxic conditions throughout pregnancy.^43^, ^44^, ^45^ The response of placental trophoblasts to hypoxia involves activating specific genes, including *PGF*,^46^ *PAPPA2*,^47^ *LEP*,^48^ *FLT1*,^49^ *CGB3*,^50^ *FSTL3*,^51^ *IGF*,^52^ and *ADAM12*.^53^ We observed higher expression levels of these genes in FGR placentas compared to AGA placentas, with fold changes (FC) ranging from 1.82 to 6.19 (Figure 4A).

Placental responses to hypoxia aim to preserve ATP levels, critical for maintaining placental functions under low oxygen conditions.^54^ KEGG‐HIF signaling pathway, which is active under hypoxia, reveals involvement of glucose metabolism (*HK1*, *HK2*, *PGM1*, *PFKL*, *PFKFB1*, *PKM*, *ALDOA*, *ENO2*, *GLUT1*), the pentose phosphate pathway (PPP) (*G6PD*, *TKT*, *TALDO*), vascular tone (*NOS3*), and angiogenesis (*VEGF*, *FLT1*). FGR placentas show stronger upregulation of these genes than AGA placentas, with FGR upregulation exceeding that in SGA (Figure 4B). Notably, RNA‐seq data show that *G6PD*, which encodes the enzyme that directs glucose‐6P to PPP, is upregulated twofold in FGR and SGA placentas compared to AGA, indicating increased PPP activity to maintain redox balance and R5P production (Figure 4C).

Metabolomic data confirmed PPP activation in FGR placentas, showing higher levels of key metabolites like fructose 6‐phosphate (F6P) and 6‐phosphogluconate (6PG) (Figure 4D). Additionally, glycerol‐3‐phosphate (Gro3P) was found significantly more abundant in FGR compared to AGA placentas. The interconversion of glyceraldehyde‐3‐phosphate (G3P) to dihydroxyacetone phosphate (DHAP), then to Gro3P, likely plays a crucial role in maintaining NAD+ homeostasis.^55^ LDHA's product, lactic acid, was also higher in FGR placentas, supporting glycolysis, steroid hormone synthesis, and phospholipid/glyceride biosynthesis within the trophoblast (Figure 4E).

Hypoxia‐induced transcriptional signaling and anaerobic metabolism are physiological adaptations to decreased oxygenation in FGR placentas, which reduce energy production and negatively affect fetal growth.^54^ We explored the metabolic adaptability of AGA and FGR placentas under normoxic and hypoxic conditions using placental organoids (PlOs). PlOs from AGA and FGR groups were cultured at 2% oxygen (mimicking placental low oxygen level present in fetal growth restriction) and 8% oxygen (mimicking term placenta normoxia). Remarkably, FGR PlOs at 2% oxygen exhibited higher ATP levels than AGA PlOs, indicating more efficient metabolic adaptation to hypoxia (Figure 4F). The data suggest that FGR PlOs maintain a hypoxic preconditioned state under normoxia, unlike AGA PlOs, which show a strongly reduced metabolic activity under hypoxia. In summary, our data highlight the activation of metabolic and transcriptional mechanisms facilitating adaptation to hypoxia in dysfunctional FGR placentas. These adaptations ensure placental metabolic functionality, albeit at a reduced level compared to AGA placentas.

### 3.6 Arginine metabolism is subverted in FGR placentas: role of creatine pathway

Arginine metabolism significantly impacts trophoblast function, influencing differentiation, invasion, and syncytiotrophoblast viability.^56^, ^57^ Dysregulated arginine metabolism is associated with conditions like FGR and PE.^58^, ^59^, ^60^ Notably, arginine supplementation has been shown to alleviate hypertension and promote fetal growth in pregnant women with hypertensive disorders and PE.^36^ Though classified as a nonessential amino acid, arginine is central to the urea cycle (UC) and serves as a substrate for synthesizing polyamines, creatine, and nitric oxide (NO).^61^

**FIGURE 5:**
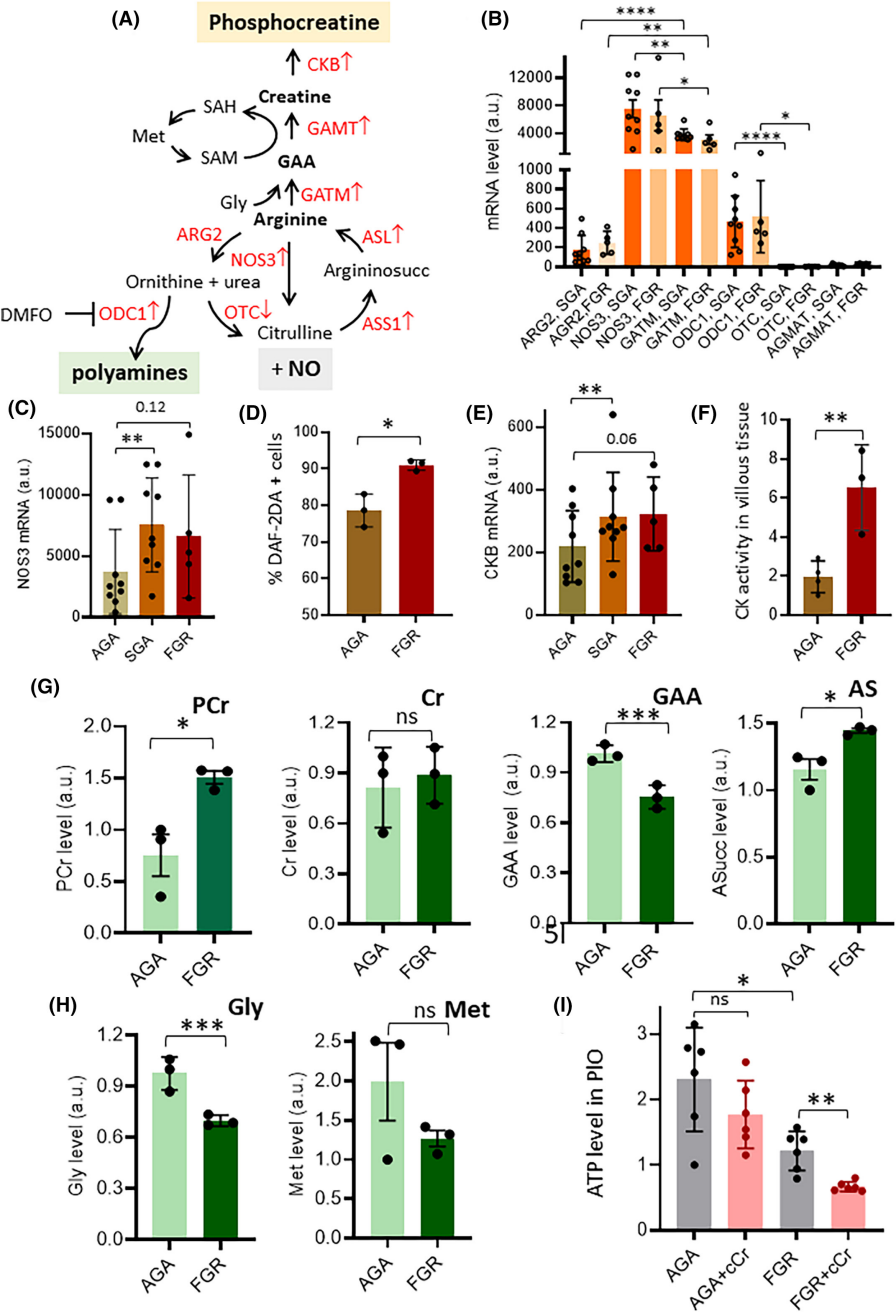
Metabolism of arginine is subverted in FGR placentas. (A) Metabolic network showing the route of arginine in placenta. Arginine can be channeled into the creatine, polyamine, or nitric oxide pathways; genes encoding key enzymes involved in arginine metabolism and found dysregulated in FGR placentas are highlighted in red. Up arrows indicate upregulation and down arrows repression. (B) Absolute mRNA levels of the indicated genes (encoding key enzymes of the arginine metabolism) in FGR/SGA placentas. Data were obtained from RNA‐seq. (C) Absolute mRNA levels of NOS3 in FGR/SGA and AGA placentas; data were obtained from RNA‐seq. (D) Percentage of cells positive to DAF‐2DA in AGA and FGR PlO kept in TOM. (E) Absolute mRNA levels of CKB mRNA in FGR/SGA and AGA placentas; data were obtained from RNA‐seq. (F) Creatine kinase activity was measured in 3 AGA and 3 FGR placental biopsies, later used to obtain organoids (FGR 1, 2, 3 and AGA 4, 5, 6 samples). (G, H) Relative levels of the indicated metabolites representing key intermediates of creatine pathway determined by mass spectrometry in AGA and FGR placentas. PCr: Phosphocreatine, Cr: Creatine, GAA: Guanidinoacetic Acid, AS: Argininosuccinic acid, Gly: Glycine, and Met: Methionine. (I) ATP levels in AGA and FGR PlOs cultured in TOM and treated or not for 72 h with Cyclocreatine (CCr, 5 mM). Data represent the mean ± SD of at least 3 independent experiments: *p ≤ .05; **p ≤ .01; and ***p ≤ .001 by Student's *t*‐test, with the exception of Figure 5I (Dunn's multiple comparison test); Figure 5C and Figure 5E (Wilcoxon signed‐rank test).

In FGR and SGA placentas, RNA‐seq analysis revealed significantly lower expression of ornithine transcarbamylase (*OTC*) compared to enzymes such as nitric oxide synthase 3 (NOS3), ornithine decarboxylase 1 (*ODC1*), arginine–glycine amidinotransferase (*GATM*), and arginase 2 (ARG2). This suggests that arginine in FGR and SGA placentas primarily fuels NO, creatine, and polyamine production rather than entering the urea cycle (UC) (Figure 5B).

NO is an essential signaling molecule with vasodilatory effects that maintain placental vascular function and ensure adequate blood flow to support fetal growth.^62^ Although *NOS3* is typically highly expressed in the placenta, its expression was even higher in FGR and SGA placentas than in AGA ones (Figure 5C). To quantify NO production, single‐cell suspensions from enzymatically dissociated AGA and FGR placental organoids (PlOs) were loaded with 10 μM 4,5‐diaminofluorescein‐2‐diacetate (DAF‐2DA).^63^ Significantly increased NO production was detected in FGR PlOs, confirming that arginine in FGR is also diverted to NO production (Figure 5D).

Arginine can also serve as a substrate for GATM, producing guanidinoacetate (GAA), the precursor for the synthesis of creatine (Figure 5A). After phosphorylation by creatine kinase B (CKB), creatine is converted to phosphocreatine, an energy‐rich compound that forms a temporal energy reserve.^64^ The formation of an energy buffer in the form of phosphocreatine may be crucial for FGR placentas to compensate for the limited capacity to generate ATP under anaerobic conditions.^59^ Although RNA‐seq shows that *CKB* is slightly more expressed in FGR than in AGA placentas, its activity in FGR was found threefold higher than in AGA (Figure 5E,F).

Metabolomic data indicate that the creatine pathway is active in FGR (Figure 5G,H). Major metabolites of the creatine pathway, such as GAA, glycine, and methionine, were ~30% less abundant in FGR villous tissue than in AGA villous tissue, while phosphocreatine was approximately twofold more abundant. To demonstrate that phosphocreatine prevents ATP depletion in FGR PlOs, we inhibited the creatine pathway in AGA and FGR placentas with cyclocreatine, a competitive inhibitor of CK.^64^ This inhibition should reduce ATP levels, as phosphocreatine + ADP ↔ creatine + ATP. Treatment with cyclocreatine indeed led to a decrease in ATP levels in FGR PlOs but not in AGA PlOs, demonstrating the critical role of the creatine‐energy pathway in FGR placentas (Figure 5I).

### 3.7 Acetylation of polyamines reduces their availability in FGR placenta

Despite similar levels of ornithine and citrulline in FGR and AGA placentas, higher levels of argininosuccinate (~26%, *p* < .05) were observed in FGR placentas. This suggests the urea cycle is not involved in metabolic adaptation in the dysfunctional FGR placenta. The increase in argininosuccinate likely results from the conversion of arginine to citrulline and NO via NOS3 and its subsequent regeneration to arginine, maintaining the levels of this critical amino acid in the FGR placenta (Figure 5A).

**FIGURE 6:**
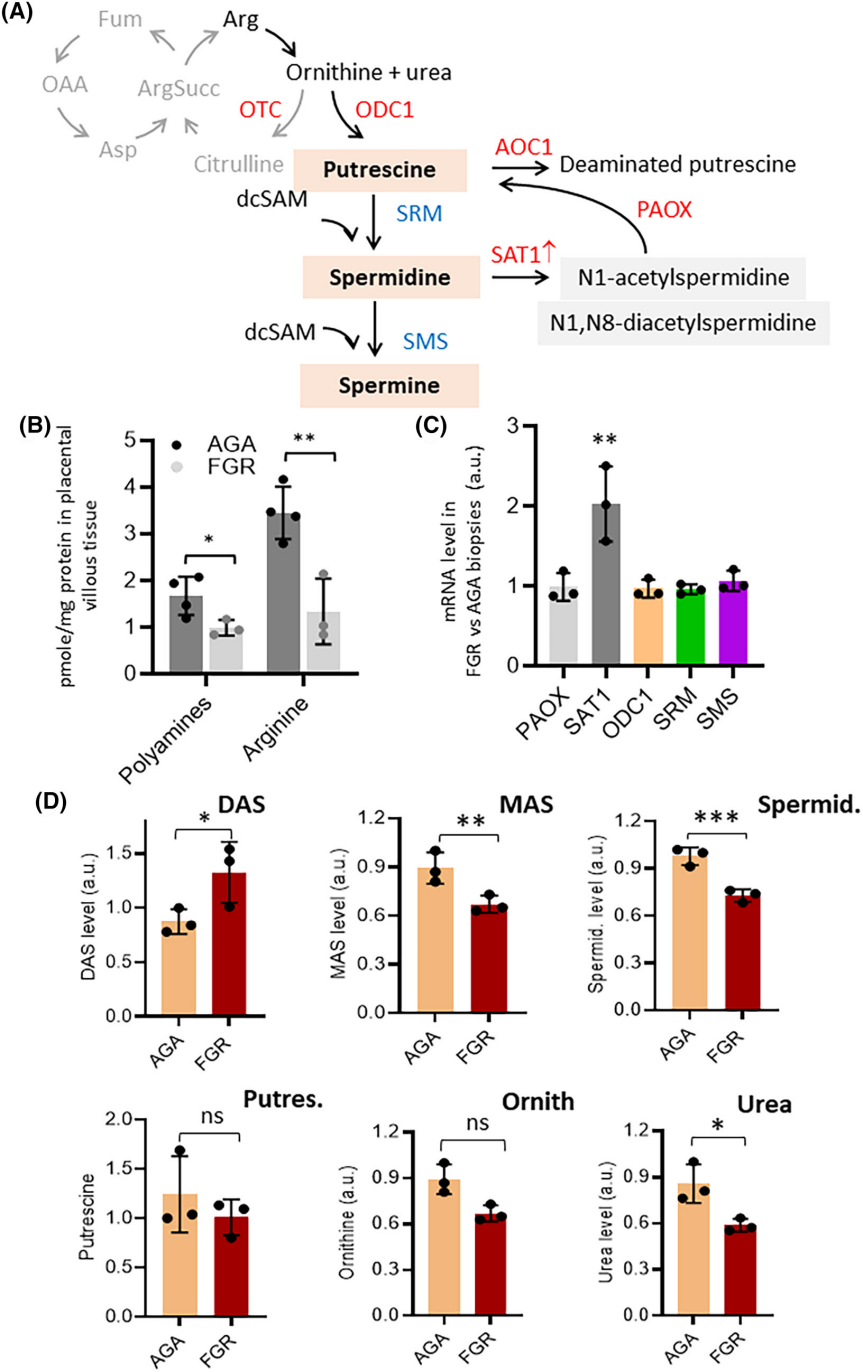
The metabolism of arginine and polyamine is altered in FGR placentas. (A) Metabolic network showing that arginine can feed the polyamine pathway. (B) Absolute levels of arginine and polyamines in AGA and FGR placental villous tissue. (C) mRNA level of genes related to arginine metabolism determined by RT‐qPCR in AGA and FGR biopsies. Data are expressed as relative expression in FGR with respect to AGA samples. (D) Relative levels of the indicated metabolites of polyamine metabolism. DAC: N1, N8‐diacetylspermidine, MAS: N1‐acetylspermidine, Spermid.: Spermidine, Putres.: Putrescine, and Ornith.: Ornithine. Data represent the mean ± SD of at least 3 independent experiments: *p ≤ .05; **p ≤ .01; and ***p ≤ .001 by Student's *t*‐test.

Arginine contributes to polyamine biosynthesis through arginase and ornithine decarboxylase (ODC1), leading to the formation of putrescine, spermidine, and spermine.^65^, ^66^ The low expression of OTC compared to ODC1 in FGR and SGA placentas (Figure 5B) favors ornithine entering the polyamine pathway rather than the urea cycle (Figure 6A). Polyamine biosynthesis supports cell growth and differentiation during normal placentation.^66^ Inhibition of ODC1 in rodents slows embryonic development and causes abnormal placental vascularization and steroidogenesis.^67^, ^68^

**FIGURE 7:**
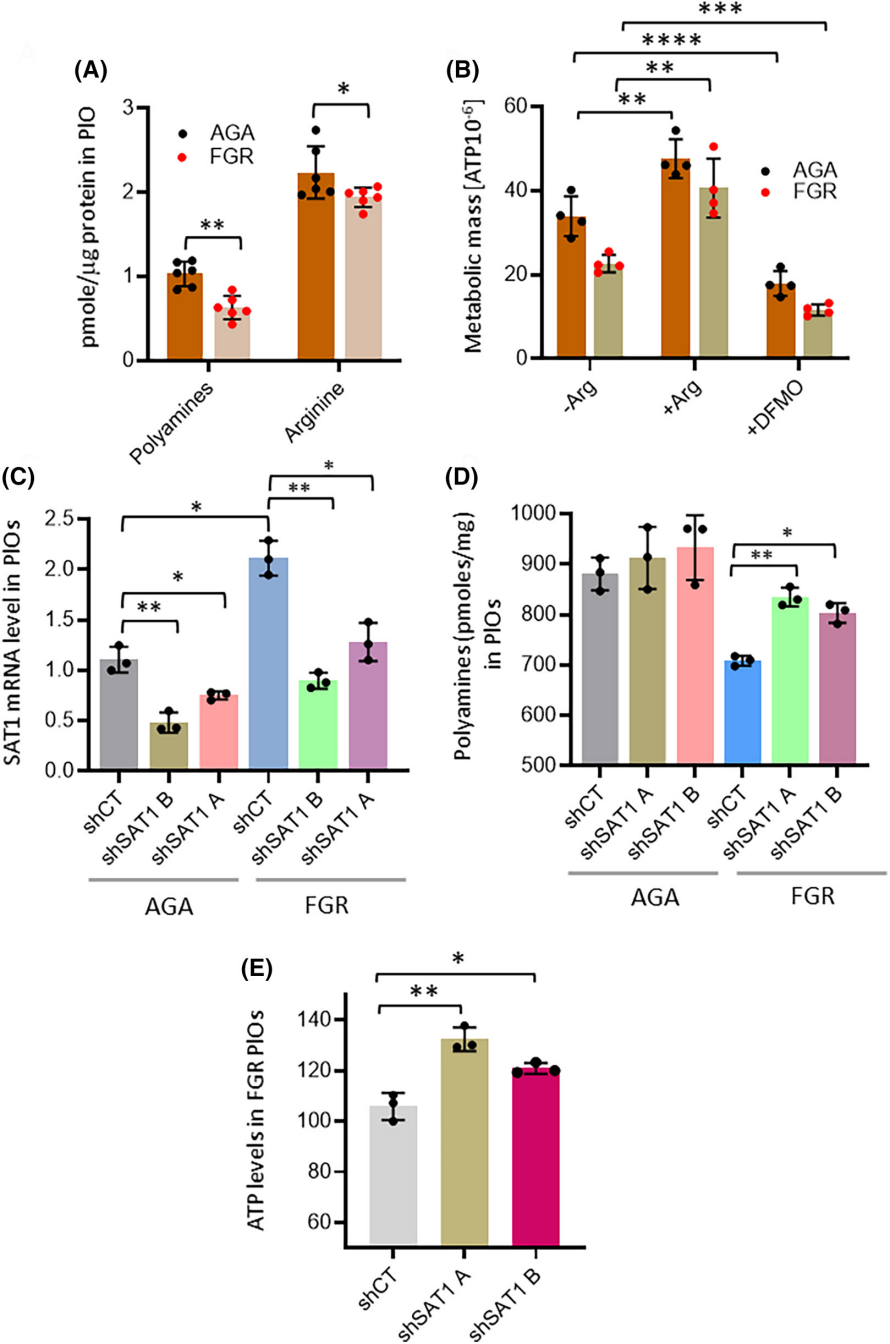
Increased spermidine acetylation depletes polyamines in FGR placentas. (A) Absolute levels of total (acetylated and diacetylated) polyamines and arginine in AGA and FGR organoids maintained for 11 days in TOM; 2 separate cultures of each of the 3 AGA and FGR PlO were analyzed. (B) Absolute levels of ATP in AGA and FGR PlOs cultured for 11 days in TOM and for the last 2 days in medium deprived of arginine or in medium containing 5 μM DFMO. (C) mRNA levels of SAT1, determined by RT‐qPCR, in AGA and FGR PlOs (AGA4 and FGR1) stably expressing the indicated two shRNA antisense to SAT1 (shSAT1A, shSAT1B) or shCT (against GFP). 3 polyclonal cultures of each PlO clone were compared. Data are relative to one culture of AGA4. (D) Absolute levels of total polyamines in AGA (PlO 4) and FGR (PlO1) organoids silenced or not for SAT1 as indicated and explained in Figure 7C. (E) ATP levels in three independent cultures of FGR organoids (PlO 1), silenced or not for SAT1 as explained in Figure 7C, after 11 days of culture in TOM after removal of puromycin. Data represent the mean ± SD of at least 3 independent experiments or cultures as indicated in the legend: *p ≤ .05; **p ≤ .01; and ***p ≤ .001 by Dunn's multiple comparison test.

FGR placentas exhibited lower levels of arginine and polyamines in villous tissue compared to AGA placentas (Figure 6B). RT‐qPCR showed no significant changes in the expression of key polyamine metabolism genes (*ODC1, SRM, SMS, PAOX*), except for spermine/spermidine N1‐acetyltransferase 1 (*SAT1*), which catalyzes the acetylation of spermine and spermidine, was twice as expressed (*p* < .01) in FGR than in AGA placentas (Figure 6C). This would decrease spermidine and N1‐acetylspermidine (MAS) levels as well as increase N1,N8‐diacetylspermidine (DAS) levels.^69^ In fact, metabolomic data confirmed decreased spermidine and MAS and increased DAS in FGR placentas compared to AGA placentas (Figure 6D). Similar results were observed in FGR PlOs, which showed lower arginine and total polyamine content compared to AGA PlOs (Figure 7A). To investigate the effects of arginine and polyamines on ATP levels, PlOs were cultured in a medium with and without arginine. Arginine deficiency resulted in lower ATP levels in both AGA and FGR PlOs (Figure 7B), likely due to suppression of the creatine pathway, an important energy source for the dysfunctional placenta. Treatment with difluoromethylornithine (DFMO), a suicide inhibitor of ODC1 that blocks polyamine synthesis,^70^ caused a significant decrease in ATP in both types of PlOs (Figure 7B), indicating that polyamines are critical for maintaining ATP levels in the dysfunctional FGR placenta.

To explore whether polyamine deficiency in FGR placentas is due to increased SAT1 expression, we suppressed *SAT1* in AGA and FGR PlOs using lentiviral particles expressing two different shRNAs (Figure 7C). This resulted in an increase in total polyamine and ATP content in FGR PlOs, suggesting partial reactivation of metabolism (Figure 7D,E). Overall, the results highlight the reprogramming of arginine metabolism in FGR placentas, with SAT1‐induced acetylation of polyamines impairing their availability for syncytiotrophoblast and cytotrophoblast function. These findings position SAT1 as a potential therapeutic target for developing specific inhibitors to treat placental dysfunction and FGR.

## 4 DISCUSSION

In this study, we identified distinct characteristics of third‐trimester placentas in FGR compared to AGA infants. Several notable features emerged in the FGR placenta: (i) hypoxic conditions, indicating inadequate oxygen supply^36^, ^71^; (ii) increased reliance on oxygen‐independent pathways, such as glycolysis and arginine/phosphocreatine, for ATP production; and (iii) altered polyamine metabolism, characterized by lower levels of spermidine/N1‐acetylspermidine and increased levels of diacetylspermidine.

When comparing the transcriptomes of FGR placentas with those from the Pregnancy Outcome Prediction (POP) study,^21^ we identified 78 differentially expressed genes (DEGs), with 38 downregulated and 40 upregulated. The downregulated genes in FGR were associated with defects in the syncytiotrophoblast basal membrane and impaired placental perfusion, while the upregulated genes were linked to hypoxia and HIF signaling pathways. These gene expression differences were further validated by qRT‐PCR analyses in AGA, FGR, and SGA groups, confirming that the transcriptional profile of SGA placentas falls midway between that of healthy AGA and pathological FGR placentas. This intermediate pattern was also observed in the expression of genes involved in glycolysis and the pentose phosphate pathway (PPP).

Our RNA‐seq data revealed a significant upregulation of hypoxia‐regulated genes, including *PAPPA2*, *LEP*, *FLT1*, *CGB3*, *FSTL3*, *IGF2*, and *ADAM12*, in dysfunctional FGR placentas compared to AGA placentas.^47^, ^48^, ^49^, ^50^, ^51^, ^52^, ^53^ The upregulation of these genes in FGR likely reflects compensatory mechanisms aimed at supporting fetal growth despite impaired placental development. GO revealed enrichment of hypoxia‐related signaling pathways, with the upregulated genes involved in glycolysis and the pentose phosphate pathway.

Given the limited energy yield of glycolysis (2 ATP per glucose molecule), the FGR placenta also relies on arginine to meet its metabolic demands. This reliance is evidenced by the lower arginine content in FGR villus tissue and organoids compared to AGA, as arginine supports bioenergetic pathways and phosphocreatine production, essential for maintaining energy homeostasis in hypoxic conditions. Metabolomic data confirmed a depletion of glycine, methionine, and guanidinoacetate (GAA), together with increased phosphocreatine levels, aligning with the conversion of arginine to phosphocreatine in the FGR placenta. Treatment with cyclocreatine, a competitive inhibitor of creatine kinase (CK), resulted in decreased ATP levels in FGR organoids, underscoring the critical role of arginine in maintaining placental energy balance. Our findings are consistent with recent studies indicating higher total creatine content (creatine plus phosphocreatine) in the third‐trimester FGR placenta compared to healthy controls, coupled with reduced GAA concentrations.^59^ Notably, our study shows that phosphocreatine and not creatine increases twofold in the FGR placenta. We also observed a significant downregulation of GAA (approximately 50%) and upregulation of genes involved in creatine metabolism (*GATM, GAMT, CK*), emphasizing active creatine metabolism as an adaptive response to hypoxia in the FGR placenta.

Furthermore, GSEA revealed that among the 762 genes depleted in the human placenta,^38^ those associated with polyamine metabolism were notably upregulated in FGR placentas. This observation aligns with results from an unbiased metabolomic analysis comparing AGA and FGR placentas, which identified differentially abundant metabolites (DAMs) in FGR placentas. GO analysis revealed that arginine and proline metabolism were among the enriched categories of upregulated DAMs. A key finding from our study is the reduction in polyamine levels and the concurrent elevation of SAT1 in FGR placentas. Further sub‐analysis of the RNA‐seq data confirmed SAT1 upregulation in FGR placental samples from both male and female newborns, while SMS expression was higher in males compared to females (Figure S7).

Polyamines play crucial roles in various biological processes, including trophoblast proliferation and placental vascularization.^72^ A recent study showed that spermine synthase, a key enzyme of polyamines metabolism, escapes X‐chromosome inactivation in the placenta, leading to increased polyamine levels in female placentas.^73^ Although our sample size is too small to confirm quantitative differences in polyamines and diacetylspermidine between female and male placentas, it shows a decline in global polyamine levels and an increase in diacetylspermidine levels in FGR placentas. Metabolomic studies indicate that polyamines regulate central energy metabolism, evidenced by decreased mRNA levels of TCA and OXPHOS enzymes following DFMO treatment.^74^ Consistently, we observed approximately a twofold reduction in ATP levels in organoids from FGR trophoblasts after treatment with the polyamine synthesis inhibitor DFMO.

Two independent studies have reported that overactivation of SAT1 correlates with the depletion of the acetyl‐CoA cellular reservoir.^75^, ^76^ While we found no evidence of an imbalance in acetyl‐CoA levels in FGR placentas, *SAT1* expression was about twofold higher in dysfunctional FGR placentas than in AGA placentas. We found that *SAT1* depletion in FGR organoids led to partial restoration of polyamine levels and ATP production. Whether *SAT1* inhibition can restore or improve placental function in FGR placentas in vivo remains to be demonstrated.

Finally, we would like to point out that the CTB, STB, and EVT trophoblasts have different metabolic profiles that reflect their specific functions during placental development.^29^, ^30^ We identified the trophoblast subpopulations in the placental biopsies and organoids and found minimal differences between AGA and FGR. However, we cannot exclude that even these subtle variations may contribute to the observed metabolic differences between AGA and FGR.

## 5 CONCLUSION

Our study has several clinically relevant implications. Firstly, it underscores the crucial role of creatine as an essential energy reservoir, facilitating ATP recycling. Given its importance in energy production, particularly in hypoxic FGR placentas, exogenous creatine holds promise for improving outcomes in growth‐restricted pregnancies. Maternal creatine supplementation has shown potential in animal studies, especially in cases of intrapartum birth asphyxia.^77^ Additionally, maternal dietary creatine supplementation during gestation has provided neuroprotective effects and benefits to other organs affected by intrapartum asphyxia.^78^ Our RNA‐seq data show a 1.7‐fold increase in the expression of the creatine transporter *SLC6A8* in FGR placentas compared to AGA placentas, suggesting an increased demand or utilization of creatine in FGR. While animal studies have shown promising neuroprotective effects of maternal creatine supplementation, there is currently a lack of published trials evaluating its use for fetal neuroprotection in humans.^79^

Secondly, our study highlights the metabolic role of arginine. Several studies have suggested that arginine supplementation shows promise as a potential treatment for FGR. Through the production of nitric oxide, arginine can improve the placental blood flow via vasodilation, thereby enhancing oxygen and nutrient supply to the fetus.^80^, ^81^, ^82^ While this strategy requires further investigation,^83^ our findings provide valuable insights into the mechanisms by which arginine improves the energetic conditions in dysfunctional FGR placentas, suggesting a preventive approach. However, given the overexpression of *SAT1*, arginine supplementation alone may not sufficiently address polyamine deficiency in dysfunctional placentas. Our study supports the development of specific SAT1 inhibitors to be used in combination with arginine to overcome polyamine deficiency in FGR placentas and potentially mitigate associated pathologies.

## AUTHOR CONTRIBUTIONS

EDG, SX, and LX designed the study; EDG, SX, and VT planned and contributed to experiments; YC performed RNA library preparation; NG supervised transcriptomic analysis; EDG, APL, and RP performed bioinformatic analyses; SX, MO, EDG, and LD contributed to samples and scientific guidance; EDG and LX contributed to funding; LX, SX, and EDG wrote and edited the manuscript; and EDG and SX contributed equally to this work. Order of co‐first authorship for publication was determined alphabetically by last name.

## FUNDING INFORMATION

This work was supported by NextGenerationEU and Ministry of University and Research Ministero dell'Università e della Ricerca (PRIN_PNRR 2022 P2022THRT3 to E.D.G) and by funds from the University of Udine to LX.

## DISCLOSURES

The authors declare no conflict of interest exists.

## ETHICS STATEMENT

The Regional Review Board and the Clinical Research Center of the Santa Maria della Misericordia Hospital approved the present study (ASUFC, approval n. 289 of March 17, 2021), which complied with the requirements of the general authorization of the Italian Data Protection Authority for the processing of data for scientific research purposes.

## Supporting information


Data S1.

## Data Availability

The RNA‐seq data in this study have been deposited in the GEO database with the accession code GSE262116, which is publicly available. Metabolomics raw and processed data have been deposited in MetaboLights with the accession code: MTBLS10236.
