# Supplementary material for: The central role of creatine and polyamines in fetal growth restriction
Source: FASEB J. 2024 Nov 30;38(23):e70222. doi: 10.1096/fj.202401946R (PMC11607630; doi:10.1096/fj.202401946R)

Supplemental data

The central role of creatine and polyamines in fetal growth restriction

Eros Di Giorgio,^1,&,^* Serena Xodo,^2,&^ Maria Orsaria,^3^ Laura Mariuzzi, ^3^ Raffaella Picco,^1^ Vanessa Tolotto,^1^ Ylenia Cortolezzis,^1^ Francesca d’Este,^1^ Nicole Grandi,^4^ Lorenza Driul,^1,2^ Ambrogio Londero,^5^ Luigi E. Xodo,^1,^*

^1^ Department of Medicine, University of Udine, 33100 Udine, Italy;

^2^ Clinic of Obstetrics and Gynecology, Santa Maria della Misericordia Hospital, ASUFC, 33100 Udine, Italy;

^3^ Institute of Pathology, Department of Medicine, University of Udine, 33100 Udine, Italy;

^4^ Laboratory of Molecular Virology, Department of Life and Environmental Sciences, University of Cagliari, 09124 Cagliari, Italy.

^5^ Obstetrics and Gynecology Unit, IRCCS Institute Giannina Gaslini, 16147 Genova, GE, Italy;

^&^ EDG and SX contributed equally to this work;

* LEX and EDG are co–corresponding authors.

Supplementary Tables

**Table S1**. List of the primers used for qRT-PCR experiments. The sequence is reported in 5’->3’;

**Tables S2A-C**. Characteristics of patients whose placental biopsies were used for the study;

**Table S2D**. Patients whose placental biopsy was used to create organoids;

**Table S3.** Bootstrapping signature of 4006 differentially expressed coding genes between our AGA and FGR. Enclosed file;

**Table S4**. Dysregulated signalling pathway: FGR down- and up-regulated genes after bootstrapping;

**Table S5**: List of 78 leading edge genes up- and down-regulated in FGR;

**Table S6** List of reported placental depleted transcripts (ref. 37) and of leading edge placental depleted transcripts enriched in FGR vs AGA;

**Table S7**. Functional enrichment analysis of metabolites found increased or decreased in FGR vs AGA;

**Table S1.** List of the primers used for qRT-PCR experiments. The sequence is reported in 5’->3’.

| Oligonucleotide name | Sequence (5'->3') | |
| --- | --- | --- |
|  |  | |
| SAT1 FW | | ACCCGTGGATTGGCAAGTTAT |
| SAT1 RV | | TGCAACCTGGCTTAGATTCTTC |
| INHA FW | | TTCCACTACTGTCATGGTGGT |
| INHA RV | | AGTGCTGCGTGAGAAGGTTG |
| FSTL3 FW | | GTGCCTCCGGCAACATTGA |
| FSTL3 RV | | GCACGAATCTTTGCAGGGA |
| CKB FW | | GCTGCGACTTCAGAAGCGA |
| CKB RV | | GGCATGAGGTCGTCGATGG |
| CKMT2 FW | | CCAAGCGCAGACTACCCAG |
| CKMT2 RV | | GGTGTCACCTTGTTGCGAAG |
| GPT FW | | GGGTTCGCAGTTCCACTCATT |
| GPT RV | | CCGCACACTCATCAGCTTCA |
| ARMCX4 FW | | ATGGGCCGCATTCAGGAAG |
| ARMCX4 RV | | GGCTCTGTACCCCAACCAC |
| ARMCX3 FW | | TCTGGGGCCAGGTATAATGAC |
| ARMCX3 RV | | GGAAGCCCGTTTCTGGACA |
| IGF1R FW | | TCGACATCCGCAACGACTATC |
| IGF1R RV | | CCAGGGCGTAGTTGTAGAAGAG |
| GAPDH FW | | CCCTTCATTGACCTCAACTACATG |
| GAPDH RV | | TGGGATTTCCATTGATGACAAGC |
| HPRT FW | | AGACTTTGCTTTCCTTGGTCAGG |
| HPRT RV | | GTCTGGCTTATATCCAACACTTCG |
| ERVW1 FW | | TGGCCCAAGATTCCATTCCT |
| ERVW1 RV | | TTGCCAAAATGTTACCGGGG |
| ERVFC1-1 FW | | TGCAGAAGACAAGGCAATG |
| ERVFC1-1 RV | | AGTGTTCCCTTGGACA |
| ERVFRD1 FW | | TCAAATGGTGCAGTGACTCG |
| ERVFRD1 RV | | GTTCTGGCTCTGGAGTTTAAGG |
| TP63_FW | | GGACCAGCAGATTCAGAACGG |
| TP63_RV | | AGGACACGTCGAAACTGTGC |
| XRCC6_FW | | GTTGATGCCTCCAAGGCTATG |
| XRCC6_RV | | CCCCTTAAACTGGTCAAGCTCTA |
| SRSF2_FW | | CCCGATGTGGAGGGTATGAC |
| SRSF2_RV | | GAGACTTCGAGCGGCTGTAG |
| CGA_FW | | TGCCCAGAATGCACGCTAC |
| CGA_RV | | TTGGACCTTAGTGGAGTGGGA |
| PSG2_FW | | GACAGTCAAAGTCTCTGCTTCTAC |
| PSG2_RV | | TCACAGCTGCTATGTTGGATTA |
| HSD3B1_FW | | CACATGGCCCGCTCCATAC |
| HSD3B1_RV | | GTGCCGCCGTTTTTCAGATTC |
| MMP2_FW | | TACAGGATCATTGGCTACACACC |
| MMP2_RT | | GGTCACATCGCTCCAGACT |
| ITGA5_FW | | GGCTTCAACTTAGACGCGGAG |
| ITGA5_RV | | TGGCTGGTATTAGCCTTGGGT |
| FSTL3_FW | | GTGCCTCCGGCAACATTGA |
| FSTL3_RV | | GCACGAATCTTTGCAGGGA |

**Table S2A**: Baseline characteristics of patients with appropriate for gestational age (AGA) fetuses whose placental biopsy was used for the study

| N° pt | Age | Maternal comorbidity | GA at delivery  (weeks) | IOL | Delivery mode | VOD or CD indication | Neonatal weight  (gr) | Neonatal gender | Apgar 5’ |
| --- | --- | --- | --- | --- | --- | --- | --- | --- | --- |
| 20 | 34 | no | 38+2 | no | OVD | Non reassuring FHR | 3206 | F | 10 |
| 21 | 37 | no | 40+0 | yes | SVD |  | 3385 | F | 10 |
| 18 | 20 | no | 38+5 | no | SVD |  | 3170 | F | 10 |
| 22 | 31 | no | 39+4 | no | CD | Cervical dilation arrest | 3495 | M | 10 |
| 10 | 31 | Idiopathic Olygohydramnios | 38+2 | yes | SVD |  | 3205 | M | 9 |
| 11 | 36 | no | 39+1 | no | SVD |  | 3122 | M | 8 |
| 17 | 33 | low platelets | 38+1 | no | SVD |  | 3268 | F | 10 |
| 12 | 24 | no | 41+3 | yes | SVD |  | 3905 | F | 9 |
| 33 | 36 | no | 39+1 | no | SVD |  | 3165 | M | 9 |

**Table S2B**: Baseline characteristics of patients with small for gestational age (SGA) fetuses whose placental biopsy was used for the study

| N° pt | Age | Maternal comorbidity | GA at delivery  (weeks) | IOL | Delivery mode | VOD or CD indication | Neonatal weight (gr) | Neonatal gender | Apgar 5’ |
| --- | --- | --- | --- | --- | --- | --- | --- | --- | --- |
| 16 | 34 | thrombophylia | 38+3 | no | SVD |  | 2995 | F | 9 |
| 15 | 36 | Previous traumatic pelvis fracture | 39+4 | no | CD | Planned CD | 2960 | F | 9 |
| 2 | 31 | no | 40+1 | no | SVD |  | 3000 | F | 10 |
| 19 | 29 | no | 40+2 | no | SVD |  | 2952 | F | 10 |
| 14 | 30 | Previous CD | 41+1 | no | SVD |  | 3135 | F | 10 |
| 13 | 27 | thrombophylia | 38+2 | no | SVD |  | 2720 | M | 10 |
| 24 | 30 | no | 40+0 | no | CD | Non reassuring FHR | 3070 | M | 9 |
| 6 | 33 | no | 40+4 | no | SVD |  | 3075 | M | 9 |
| 7 | 30 | no | 40+2 | no | SVD |  | 3025 | M | 10 |

**Table S2C**: Baseline characteristics of patients with growth restricted fetuses (FGR) whose placental biopsy was used for the study

| N° pt | Age | Maternal comorbidity | GA at delivery  (weeks) | IOL | Delivery mode | VOD or CD indication | Neonatal weight (gr) | Neonatal gender | Apgar 5’ |
| --- | --- | --- | --- | --- | --- | --- | --- | --- | --- |
| 8 | 34 | Chronic gastritis | 41+3 | yes | OVD | Non reassuring FHR | 2870 | M | 8 |
| 1 | 31 | no | 40+0 | yes | SVD |  | 2720 | F | 9 |
| 3 | 24 | Post-term pregnancy | 41+3 | yes | OVD | Non reassuring FHR | 2920 | F | 9 |
| 4 | 34 | no | 37+5 | yes | SVD |  | 2350 | M | 9 |
| 5 | 32 | no | 37+1 | yes | SVD |  | 2300 | M | 9 |

**Table S2D**: Baseline characteristics of patients whose placental biopsy was used to create organoid.

| N° pt | Age | Maternal comorbidity | GA at delivery  (weeks) | IOL | Delivery mode | Fetal growth | Neonatal weight (gr) | Neonatal gender | Apgar 5’ |
| --- | --- | --- | --- | --- | --- | --- | --- | --- | --- |
| 1 | 32 | obesity | 38+6 | yes | SVD | FGR | 2350 | M | 10 |
| 2 | 40 | no | 35+3 | no | SVD | FGR | 2378 | F | 10 |
| 3 | 22 | no | 37+5 | yes | SVD | FGR | 2416 | M | 9 |
| 4 | 22 | Gestational diabetes * | 38+4 | no | SVD | AGA | 3956 | M | 9 |
| 5 | 33 | no | 36+4 | no | CD | AGA | 2860 | F | 10 |
| 6 | 41 | Gestational hypertension** | 38+6 | yes | SVD | AGA | 3100 | M | 9 |

GA: gestational age

IOL: induction of labor

OVD: operative vaginal delivery

SVD: spontaneous vaginal delivery

CD: cesarean delivery

FGR: fetal growth restriction

AGA: appropriate for gestational age

FHR: fetal heart rate

PROM: premature rupture of fetal membranes

F: female

M: male

*Gestational diabetes was in good compensation under nutrition therapy alone

**Gestational hypertension was characterized by borderline values of arterial pressure, not requiring pharmacological therapy

**Table S3**. Bootstrapping identified a signature of 4006 differentially expressed coding genes between our AGA and FGR sample groups. See enclosed file (Table S3);

**Table S4.** Dysregulated signalling pathway: FGR down- and up-regulated genes after bootstrapping.

**Table S5**: List of the 78 genes (38 downregulated and 40 upregulated in FGR vs AGA), contributing to the core enrichment of GSEA, that best represents a common signature of the FGR condition.

| **Leading edge genes up-regulated in FGR** | **Leading edge genes down-regulated in FGR** |
| --- | --- |
| ENG | IL1RL1 |
| GFPT2 | EPHA3 |
| FBLIM1 | ARMCX3 |
| MICAL3 | CD44 |
| FN1 | RDH10 |
| GFOD2 | WNT5A |
| SIGLEC6 | RAPH1 |
| SYNJ2 | SLC38A2 |
| PLIN2 | IL33 |
| PROCR | NREP |
| TGFB1 | C7 |
| INHBA | NR1D2 |
| PVR | AGPAT5 |
| SYDE1 | POSTN |
| BHLHE40 | DDX60 |
| AIF1L | CLDN1 |
| MXI1 | PCDH11X |
| FLNB | PARM1 |
| HTRA4 | GCNT4 |
| CRH | OLR1 |
| SERPINB9 | NABP1 |
| AOC1 | MYO1B |
| AFAP1 | SLC22A3 |
| FSTL3 | IGF1R |
| SLC7A5 | B4GALT1 |
| RDH13 | APOD |
| NDRG1 | HECW2 |
| HSPG2 | STON2 |
| LEP | F5 |
| LYN | ADCY7 |
| COL17A1 | USP46 |
| INHA | SLC39A14 |
| ANXA4 | SLC17A5 |
| EBI3 | GBP3 |
| HTRA1 | HEG1 |
| KRT19 | COL6A3 |
| SFXN3 | ERVMER34-1 |
| PAPPA2 |  |
| SH3BP5 |  |
|  |  |

**Table S6.** List of reported placental depleted transcripts and of leading edge placental depleted transcripts enriched in FGR vs AGA.

| **Placenta depleted transcripts (Gong et al, Cell reports 2023)** | **Leading edge placental depleted transcript**  **enriched in FGR vs AGA comparison** |
| --- | --- |
| PRSS2 | CCS |
| PRSS1 | MRPL52 |
| CHL1 | MAPK8IP1 |
| CSDC2 | ENKD1 |
| MYH7 | CKB |
| PLP1 | CEL |
| FAM229A | CCDC107 |
| NPTX2 | ANKRD13B |
| TPGS1 | RIN1 |
| NOVA1 | UQCRHL |
| KIAA1755 | CCDC124 |
| MASP2 | BGLAP |
| CCDC85B | DDT |
| NAP1L2 | DPM3 |
| HEPH | METRN |
| CKM | HCFC1R1 |
| MMP17 | FAM50B |
| MB | ASPSCR1 |
| MZT2A | RABAC1 |
| KRT5 | NABP2 |
| SPRN | NELFB |
| SMPD3 | SPRN |
| SLC26A1 | ATP6V0C |
| NOXA1 | ENDOG |
| FBXL15 | MT1X |
| LDOC1 | EMC9 |
| MZT2B | THAP7 |
| RASD1 | MYH7 |
| RNF208 | ALDOC |
| MLXIPL | KRT5 |
| PDZD4 | SPTBN4 |
| CYS1 | MB |
| ZFPM1 | MRPS26 |
| TMEM160 | NT5C |
| FAM78B | ALDH1L1 |
| C16orf45 | CYS1 |
| GRIK5 | EEF2KMT |
| MRPL41 | GPT |
| FAM173A | MAMLD1 |
| SPTBN4 | PLIN1 |
| DBNDD1 | NOC4L |
| PPDPF | HSPB6 |
| ABHD17A | C17orf49 |
| CCDC107 | GSTM5 |
| NPIPB12 | STUB1 |
| PLXNB3 | MRPL12 |
| LRP3 | SEPTIN5 |
| ALDH1L1 | MRPL34 |
| TNFRSF6B | PARD6A |
| PLIN4 | RUSC2 |
| GFAP | MYPOP |
| POMC | PPAN |
| DGCR6L | LRRC37A2 |
| ZNF579 | CYP2E1 |
| IL34 | RNASEH2C |
| GFRA1 | AHNAK2 |
| GSTM5 | RPP21 |
| CPLX1 | FTSJ1 |
| PTCH2 | CTIF |
| IGFBP6 | POMC |
| ACTA1 | IL34 |
| OLFM1 | NRGN |
| RAMP1 | HABP4 |
| ENDOG | CKMT2 |
| ITGA7 | PORCN |
| GPT |  |
| C9orf16 |  |
| ALKBH7 |  |
| NME3 |  |
| HES4 |  |
| SLC51A |  |
| C19orf60 |  |
| DUSP2 |  |
| IDUA |  |
| ATP5D |  |
| ALB |  |
| LRFN4 |  |
| JOSD2 |  |
| NMNAT3 |  |
| UBTD1 |  |
| C20orf27 |  |
| HES6 |  |
| ISOC2 |  |
| NPIPA5 |  |
| ANKRD29 |  |
| PKD1 |  |
| ZFYVE28 |  |
| METTL26 |  |
| FGFRL1 |  |
| GLI4 |  |
| CHRDL1 |  |
| ZNHIT2 |  |
| KRT4 |  |
| SLC16A9 |  |
| SRPK3 |  |
| NPIPA1 |  |
| TMEM47 |  |
| ARHGAP33 |  |
| CHST1 |  |
| LIME1 |  |
| S100B |  |
| RAB3A |  |
| NSMF |  |
| CKB |  |
| ZNF219 |  |
| CCDC39 |  |
| METRN |  |
| PNPLA7 |  |
| HIC1 |  |
| MAP1LC3A |  |
| NDUFS7 |  |
| C6orf226 |  |
| SNTA1 |  |
| RCAN2 |  |
| SLC25A23 |  |
| PAOX |  |
| CH507-9B2.3 |  |
| BAHCC1 |  |
| C19orf70 |  |
| MT1X |  |
| MOSPD3 |  |
| TRAPPC5 |  |
| DNLZ |  |
| MAP3K10 |  |
| RPUSD1 |  |
| DPP7 |  |
| FAM20C |  |
| NPIPB13 |  |
| WASF3 |  |
| CCDC106 |  |
| MIB2 |  |
| C2orf16 |  |
| KRT13 |  |
| LRFN3 |  |
| PPP1R16A |  |
| TSNARE1 |  |
| C16orf86 |  |
| NCAM1 |  |
| SELENOM |  |
| DECR2 |  |
| CACNB2 |  |
| SCNN1D |  |
| SCAND1 |  |
| ZNF865 |  |
| GNAO1 |  |
| BHMT2 |  |
| ZNF688 |  |
| RNF126 |  |
| TMEM161A |  |
| FBXO44 |  |
| C11orf96 |  |
| ZNF358 |  |
| TRIM47 |  |
| RFNG |  |
| MVD |  |
| RELN |  |
| UQCRHL |  |
| SELENOO |  |
| STUB1 |  |
| ZNF787 |  |
| CLPP |  |
| TENM1 |  |
| TMEM246 |  |
| PPARGC1A |  |
| NAT6 |  |
| TBC1D3L |  |
| GAMT |  |
| PLIN1 |  |
| MFSD10 |  |
| ABLIM2 |  |
| AGT |  |
| CYP27A1 |  |
| PIDD1 |  |
| DGKQ |  |
| RHPN1 |  |
| AC138969.4 |  |
| SERPINA3 |  |
| BRAT1 |  |
| FLYWCH2 |  |
| ALDOC |  |
| BOP1 |  |
| KLHL26 |  |
| C9orf172 |  |
| MICALL2 |  |
| FNDC4 |  |
| HSPBP1 |  |
| ANKRD35 |  |
| FAAP20 |  |
| C7orf50 |  |
| C8orf82 |  |
| PLPPR2 |  |
| EXD3 |  |
| DUSP23 |  |
| ADCK5 |  |
| PRSS53 |  |
| SNED1 |  |
| CDH23 |  |
| MYH7B |  |
| NOV |  |
| KLHL25 |  |
| DBP |  |
| CISD3 |  |
| NT5M |  |
| C21orf33 |  |
| NMB |  |
| RPL13 |  |
| POLRMT |  |
| ALDH2 |  |
| MYL5 |  |
| SLC2A4RG |  |
| TSPOAP1 |  |
| SLCO3A1 |  |
| DNPH1 |  |
| ZNF628 |  |
| XRCC3 |  |
| OPLAH |  |
| RPS19BP1 |  |
| NUBP2 |  |
| ZFP41 |  |
| SRM |  |
| PYCRL |  |
| PRRT3 |  |
| SOX18 |  |
| PLIN5 |  |
| SHARPIN |  |
| TDRD6 |  |
| PQLC1 |  |
| SMOX |  |
| LMF1 |  |
| AGAP9 |  |
| C1orf122 |  |
| NPIPB11 |  |
| RAI1 |  |
| BAIAP2L2 |  |
| PIGQ |  |
| TBX6 |  |
| SSBP4 |  |
| NOC4L |  |
| DTX1 |  |
| C19orf24 |  |
| WDR18 |  |
| ZDHHC8 |  |
| NUDT8 |  |
| MYRIP |  |
| FBXW5 |  |
| CST3 |  |
| PLA2G4B |  |
| GALK1 |  |
| CROCC |  |
| FAM50B |  |
| ABCA2 |  |
| IZUMO4 |  |
| C21orf2 |  |
| FAHD2B |  |
| CKMT2 |  |
| TRMT61A |  |
| C1orf159 |  |
| LIN7B |  |
| ADAMTS13 |  |
| PCBP4 |  |
| NLGN2 |  |
| NDUFS8 |  |
| GADD45GIP1 |  |
| ABHD14A |  |
| LMF2 |  |
| H1FX |  |
| KLHL17 |  |
| DVL1 |  |
| CEP131 |  |
| UCHL1 |  |
| NFIC |  |
| AKR1C1 |  |
| NR1D1 |  |
| NPDC1 |  |
| NUMBL |  |
| TELO2 |  |
| CAND2 |  |
| RBM38 |  |
| LRRC37A2 |  |
| NRBP2 |  |
| QTRT1 |  |
| DRC3 |  |
| AKR1C2 |  |
| PTPRS |  |
| CCHCR1 |  |
| CADM4 |  |
| DOHH |  |
| PELI3 |  |
| FKBP8 |  |
| WIPF3 |  |
| SMIM10L2B |  |
| PPP1R35 |  |
| POMGNT2 |  |
| CTU2 |  |
| LRRC75B |  |
| AK1 |  |
| E4F1 |  |
| ZNF414 |  |
| PRADC1 |  |
| FTH1 |  |
| NDUFB7 |  |
| INPP5E |  |
| NPTXR |  |
| DPM3 |  |
| DMKN |  |
| RNF166 |  |
| FAAP100 |  |
| MPG |  |
| ZFHX4 |  |
| AP5Z1 |  |
| MEGF6 |  |
| MAPK8IP1 |  |
| ANKS3 |  |
| TSR3 |  |
| GSTM4 |  |
| SORD |  |
| NT5C |  |
| CHTF18 |  |
| CAPN15 |  |
| RABAC1 |  |
| SLC25A42 |  |
| SPATA20 |  |
| ZNF213 |  |
| CSNK1G2 |  |
| PARD6A |  |
| SAT2 |  |
| PIN1 |  |
| TRABD |  |
| BTBD2 |  |
| KLHL41 |  |
| NPHP4 |  |
| TSSK3 |  |
| ROBO3 |  |
| IRF2BP1 |  |
| CHRD |  |
| MIGA2 |  |
| ENO2 |  |
| VAMP1 |  |
| NME4 |  |
| TBL3 |  |
| RIN1 |  |
| VEGFD |  |
| EPN1 |  |
| HCFC1R1 |  |
| MYBBP1A |  |
| UBXN6 |  |
| DAGLA |  |
| MAP2K2 |  |
| CARNS1 |  |
| CIC |  |
| HEXDC |  |
| NCLN |  |
| TMEM134 |  |
| ANO8 |  |
| SDHAF1 |  |
| ZNF771 |  |
| ROM1 |  |
| ENKD1 |  |
| THAP7 |  |
| LRRC20 |  |
| SORCS2 |  |
| UBE2S |  |
| MAPK8IP3 |  |
| HSPB6 |  |
| NPEPL1 |  |
| ARMC5 |  |
| OTUD7A |  |
| SLC27A5 |  |
| DHRS4L2 |  |
| ATAD3A |  |
| ECI1 |  |
| CNTFR |  |
| LRRN2 |  |
| TSPYL2 |  |
| ACSF3 |  |
| SCLY |  |
| SLC2A6 |  |
| MACROD1 |  |
| MFSD3 |  |
| NTHL1 |  |
| MEF2B |  |
| ATP6V0E2 |  |
| SLC27A1 |  |
| MRPL38 |  |
| MDGA1 |  |
| SLC9A3R2 |  |
| COL4A3 |  |
| MZF1 |  |
| GNB1L |  |
| AGAP6 |  |
| PPP1R37 |  |
| DCXR |  |
| SSC5D |  |
| ANKRD13B |  |
| GPX1 |  |
| SLC52A2 |  |
| AZIN2 |  |
| LRWD1 |  |
| PTRH1 |  |
| MXD3 |  |
| MRPL4 |  |
| CDIP1 |  |
| NDUFAF3 |  |
| TMEM175 |  |
| AURKAIP1 |  |
| SLC45A3 |  |
| ST6GALNAC4 |  |
| MVB12A |  |
| C17orf49 |  |
| MROH7 |  |
| PEMT |  |
| PLEKHH3 |  |
| RPS15 |  |
| TMEM38A |  |
| ZMIZ2 |  |
| ITGA8 |  |
| CYHR1 |  |
| VPS51 |  |
| PACS2 |  |
| GTPBP6 |  |
| ANXA9 |  |
| TTLL12 |  |
| AATK |  |
| ZNF653 |  |
| MED16 |  |
| NCS1 |  |
| NDUFAF8 |  |
| ATAD3B |  |
| PDE1B |  |
| ZNF511 |  |
| WIZ |  |
| MON1A |  |
| MRPS34 |  |
| MRPL55 |  |
| PUSL1 |  |
| IRF7 |  |
| PLCD4 |  |
| TMEM223 |  |
| CAPN3 |  |
| KLHL21 |  |
| NFKBIL1 |  |
| AHDC1 |  |
| STEAP2 |  |
| RPUSD3 |  |
| TSPO |  |
| POLR2J |  |
| FPGS |  |
| KLF15 |  |
| DNAJC4 |  |
| PODXL2 |  |
| FAM127B |  |
| DDX51 |  |
| SMO |  |
| LRRC17 |  |
| MRPS24 |  |
| SCRIB |  |
| TWF2 |  |
| ATP6V0C |  |
| GOLGA8B |  |
| SNAPC2 |  |
| HSF4 |  |
| CPTP |  |
| THBS2 |  |
| NCOR2 |  |
| SAC3D1 |  |
| GLB1L2 |  |
| FBXL6 |  |
| SAMD1 |  |
| KCNAB2 |  |
| YDJC |  |
| ECSIT |  |
| RITA1 |  |
| RNASEH2C |  |
| MARK4 |  |
| GLTSCR2 |  |
| CARD19 |  |
| HSF1 |  |
| COQ8B |  |
| GAS2L1 |  |
| CYP2E1 |  |
| NUDT17 |  |
| MIIP |  |
| VKORC1 |  |
| FDX2 |  |
| SIVA1 |  |
| GUK1 |  |
| DENND6B |  |
| ZGLP1 |  |
| SNX21 |  |
| SLC26A10 |  |
| COMTD1 |  |
| SPPL2B |  |
| MT1M |  |
| FAHD2A |  |
| C12orf10 |  |
| HGH1 |  |
| SAP25 |  |
| NR4A1 |  |
| TEF |  |
| ULK3 |  |
| MFSD7 |  |
| PER1 |  |
| SIPA1L3 |  |
| DGAT1 |  |
| GPS1 |  |
| HMCN2 |  |
| PTMS |  |
| HSD11B1L |  |
| MXD4 |  |
| C19orf25 |  |
| ZNF444 |  |
| TMEM42 |  |
| A1BG |  |
| RNF187 |  |
| TIGD5 |  |
| ATOH8 |  |
| SERGEF |  |
| APRT |  |
| NAALADL1 |  |
| TMEM86B |  |
| ZNF205 |  |
| EPHX1 |  |
| MYPOP |  |
| PDCD2L |  |
| CACTIN |  |
| CYGB |  |
| NPRL3 |  |
| SHC2 |  |
| GALNT16 |  |
| FAM207A |  |
| CAPN5 |  |
| PRR12 |  |
| MARCHF2 |  |
| LAGE3 |  |
| LRRC45 |  |
| SPTBN5 |  |
| TMEM143 |  |
| STK25 |  |
| C17orf97 |  |
| KAT2A |  |
| ZNF747 |  |
| CLUH |  |
| GGT5 |  |
| SMN2 |  |
| AGRN |  |
| ASB13 |  |
| ZNF446 |  |
| PSD |  |
| ZBTB45 |  |
| C4A |  |
| PLEKHB1 |  |
| SGSM2 |  |
| APBA3 |  |
| CAPN10 |  |
| SURF2 |  |
| WDR24 |  |
| VPS28 |  |
| B9D1 |  |
| MRPL28 |  |
| ZNF775 |  |
| CEP170B |  |
| C6orf1 |  |
| UPK3BL |  |
| ACD |  |
| SELENOH |  |
| NUDT6 |  |
| CTB-50L17.10 |  |
| MRPS26 |  |
| G0S2 |  |
| PLEKHJ1 |  |
| SLC39A3 |  |
| IL17RC |  |
| PLLP |  |
| MUSTN1 |  |
| POU6F1 |  |
| MRPL12 |  |
| MPZ |  |
| SHISA4 |  |
| TST |  |
| H2AFY2 |  |
| COL9A3 |  |
| UBALD1 |  |
| DYRK1B |  |
| FBXO17 |  |
| SPATA2L |  |
| OGFR |  |
| FBXO6 |  |
| EPDR1 |  |
| GPRC5C |  |
| TMEM9 |  |
| ARVCF |  |
| FBXW9 |  |
| RUVBL2 |  |
| ADCY1 |  |
| MELTF |  |
| TCAP |  |
| ST6GALNAC6 |  |
| CCDC124 |  |
| GPI |  |
| RHOT2 |  |
| TSC22D4 |  |
| EMC9 |  |
| ZBTB17 |  |
| DHRS11 |  |
| TRPT1 |  |
| MRNIP |  |
| RAB40C |  |
| ACOT8 |  |
| MCAT |  |
| MLST8 |  |
| SPSB3 |  |
| FBF1 |  |
| CRACR2B |  |
| SMTN |  |
| PLAC9 |  |
| FTSJ1 |  |
| ASPSCR1 |  |
| CACNB1 |  |
| FAM162A |  |
| MRPL52 |  |
| CTU1 |  |
| KIFC2 |  |
| ANAPC2 |  |
| GOLGA8A |  |
| NELFA |  |
| BRF1 |  |
| SLC25A10 |  |
| ZNF48 |  |
| SCARF2 |  |
| MMP24-AS1 |  |
| DBN1 |  |
| NCKAP5L |  |
| ZNF524 |  |
| RABL2A |  |
| C1orf35 |  |
| ANKRD16 |  |
| CRTC1 |  |
| NELFB |  |
| NABP2 |  |
| CEL |  |
| EXOSC4 |  |
| TNK2 |  |
| BRICD5 |  |
| BGLAP |  |
| MUM1 |  |
| CAMK1 |  |
| FBXL14 |  |
| PORCN |  |
| KHK |  |
| TMEM201 |  |
| TPRN |  |
| ZBTB47 |  |
| GPSM1 |  |
| NCKIPSD |  |
| LINC00116 |  |
| HABP4 |  |
| CTIF |  |
| DUS1L |  |
| NARFL |  |
| DDT |  |
| CC2D1A |  |
| TNRC6C |  |
| AHNAK2 |  |
| AXIN1 |  |
| ACAP3 |  |
| FASTK |  |
| CFD |  |
| COQ10A |  |
| ARL6IP4 |  |
| PARP10 |  |
| SMARCD3 |  |
| POLD2 |  |
| LRRC14 |  |
| KLHL13 |  |
| NCDN |  |
| NHP2 |  |
| MRPL34 |  |
| CCS |  |
| FOXP4 |  |
| TMEM147 |  |
| ZDHHC11B |  |
| TSEN54 |  |
| SNRNP25 |  |
| RAB40B |  |
| THAP8 |  |
| ALDH3A2 |  |
| WBP1 |  |
| ACAP1 |  |
| MAP1S |  |
| TRAPPC12 |  |
| NEIL1 |  |
| CYC1 |  |
| ZNF584 |  |
| KCND3 |  |
| RARRES2 |  |
| HDHD5 |  |
| BORCS6 |  |
| LHPP |  |
| C16orf95 |  |
| MPV17L |  |
| TMEM219 |  |
| NFIX |  |
| KCNB1 |  |
| USP20 |  |
| GPAA1 |  |
| GAS6 |  |
| ZC4H2 |  |
| MAMLD1 |  |
| CFAP69 |  |
| FLYWCH1 |  |
| P3H3 |  |
| RYR3 |  |
| SLC25A27 |  |
| RPP21 |  |
| R3HCC1 |  |
| NRIP2 |  |
| TMOD1 |  |
| MED25 |  |
| RNF180 |  |
| AGAP3 |  |
| LRRC4B |  |
| HSD3B7 |  |
| TNS2 |  |
| NRGN |  |
| INTS1 |  |
| DACT3 |  |
| SHF |  |
| SEPTIN5 |  |
| MAP3K14 |  |
| PCYT2 |  |
| TNFSF12 |  |
| PPAN |  |
| FAM189B |  |
| RUSC2 |  |
| PNPLA6 |  |
| MRPS12 |  |
| KCNN3 |  |
| NECAB3 |  |
| TMEM259 |  |
| EMCN |  |
| COX5B |  |
| NR4A2 |  |
| AGPAT2 |  |
| TRIM65 |  |
| ASMTL |  |
| NACC2 |  |
| SNAPC4 |  |
| MT-ND6 |  |
| HLA-F |  |
| EEF2KMT |  |
| ADAMTS17 |  |
| CSTB |  |
| SBF1 |  |
| GABBR1 |  |
| SLC39A4 |  |
| MGMT |  |
| HOMER3 |  |
| TCF7L1 |  |
| TMEM129 |  |
| DNHD1 |  |
| PXMP2 |  |
| RNF122 |  |
|  |  |

**Table S7.** Functional enrichment of metabolites found increased or decreased in FGR vs AGA comparison.

| **Up-regulated metabolites (FGR vs AGA)** | **p-value** |  |
| --- | --- | --- |
| Nicotinate and nicotinamide metabolism | 0.00072 |  |
| Glycerophospholipid metabolism | 0.0095 |  |
| Arginine and proline metabolism | 0.014 |  |
|  |  |  |
| **Down-regulated metabolites (FGR vs AGA)** | **p-value** |  |
| Steroid biosynthesis | 0.00028 |  |
| Taurine and hypotaurine metabolism | 0.011 |  |
| Glutathione metabolism | 0.032 |  |
|  |  |  |

**Figure S1:** mRNA levels of nine markers that define the specific states of CTB (TP63, XRCC6, SRSF2), STB (CGA, PSG2, HSD3B1), and EVT (MMP2, ITGA5, FSTL3). These markers were identified from: (i) single-cell RNA sequencing of human blastocysts differentiating into CTB, STB, and EVT (West et al., PNAS 2019); (ii) single-cell RNA sequencing of 70,000 cells from first-trimester placental tissue (Vento-Tormo et al., Nature 2018), and were later applied to identify CTB, STB, and EVT subpopulations in placental organoids (Yang et al., eLife 2022), as well as to assess successful CTB differentiation into STB and EVT (Jeyarajah et al., PNAS 2022).

**
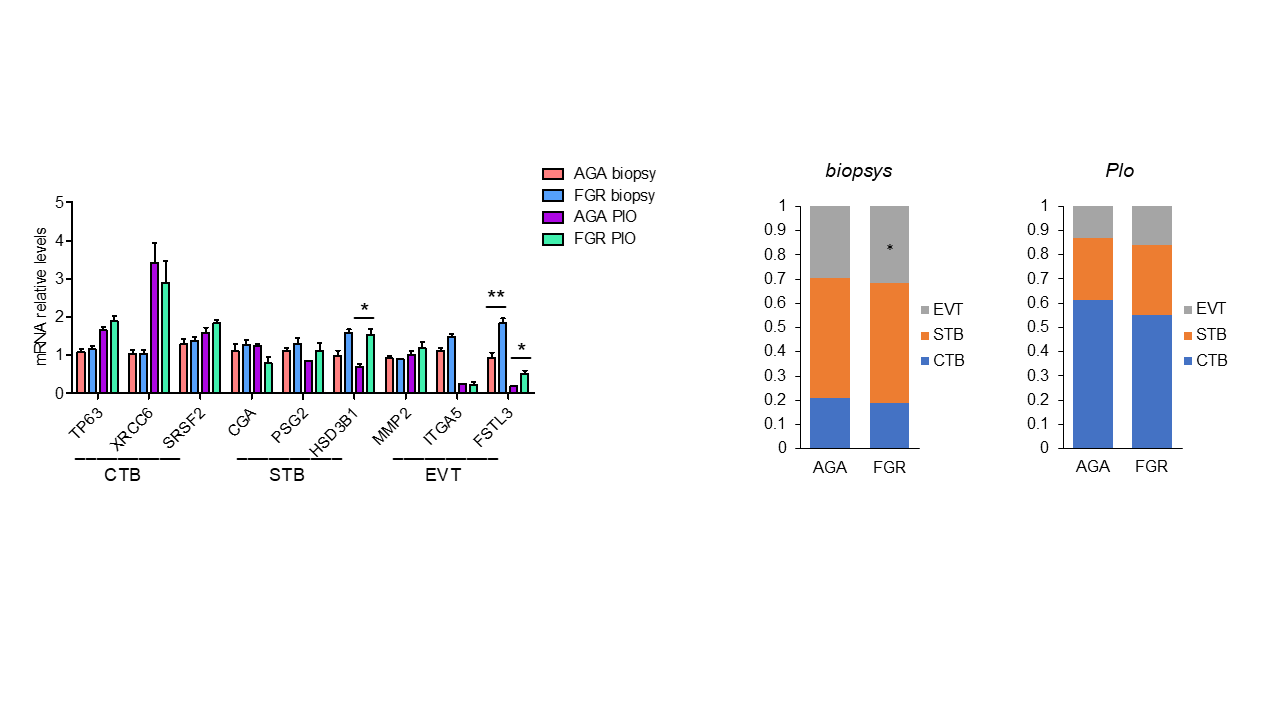
**

**Figure S2A**: heatmap of the expression levels (log2 FC) of the 182 coding genes repressed in FGR placentas in AGA, SGA and FGR placentas with respect to the mean levels detected in AGA samples.


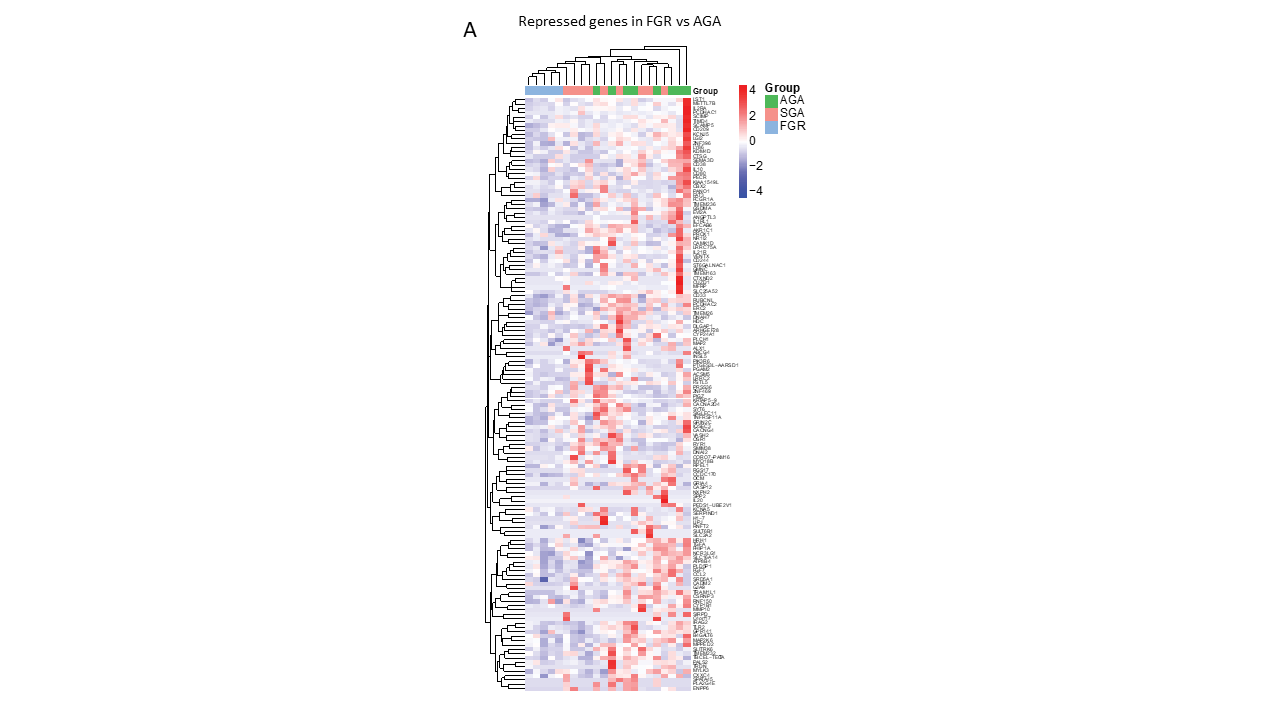


**Figure S2B**: heatmap of the expression levels (log2 FC) of the 67 coding genes up-regulated in FGR placentas in AGA, SGA and FGR placentas with respect to the mean levels detected in AGA samples.


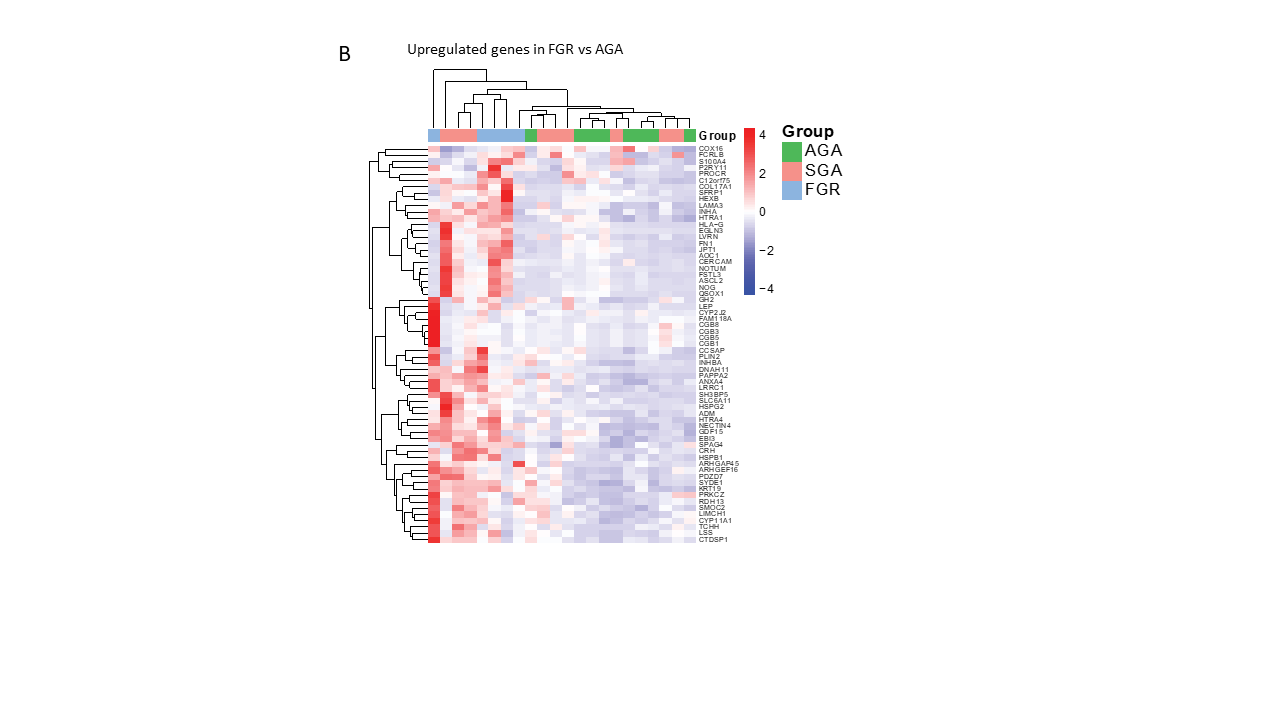


**Figure S2C**: Bootstrapping successfully segregated AGA and FGR samples. **.** PCA of the indicated samples based on the signature reported in Table S5. F=FGR, S=SGA, A=AGA.


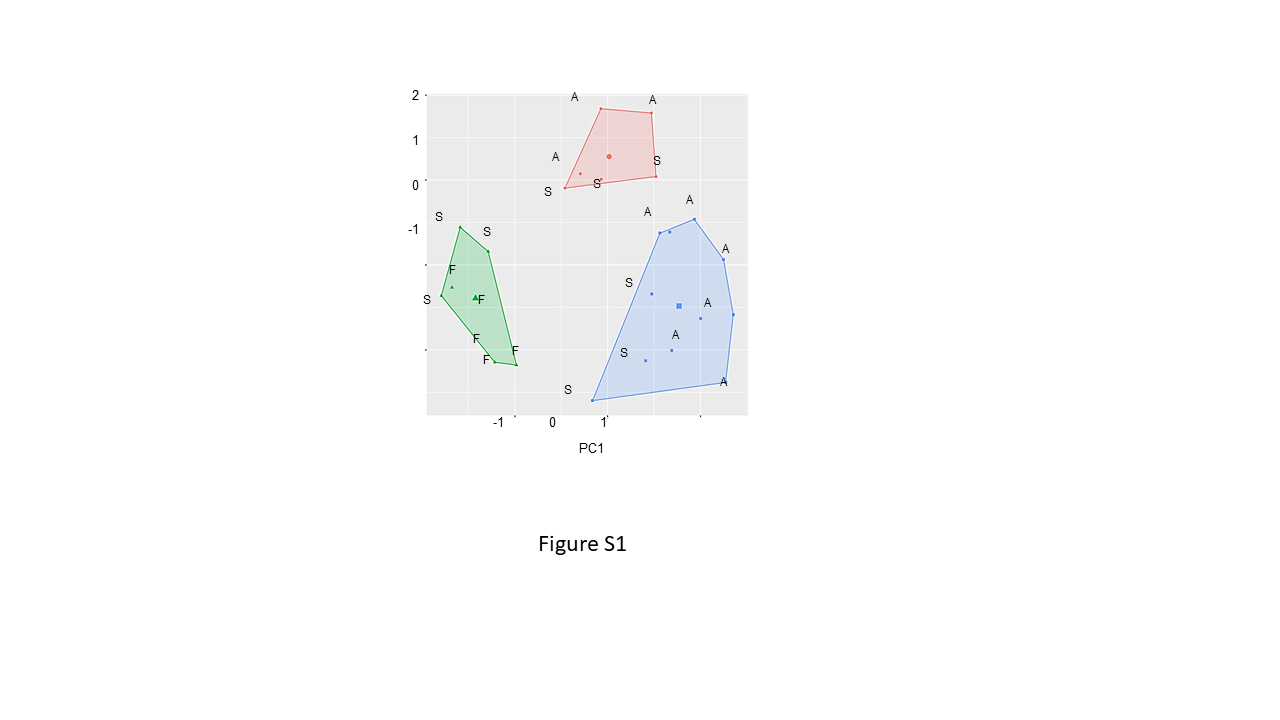


**Figure S3. A**. PCA of the indicated samples based on the signature reported in Table S4 (38 genes down-regulated in FGR). F=FGR, S=SGA, A=AGA. **B.** Heatmap of the expression levels (log2(Fc)) of the indicated genes in AGA, SGA and FGR samples in respect to the mean levels detected in AGA samples. **C.** Functional enrichment of the 38 gene signature obtained with Enrichr (<https://maayanlab.cloud/Enrichr/>).


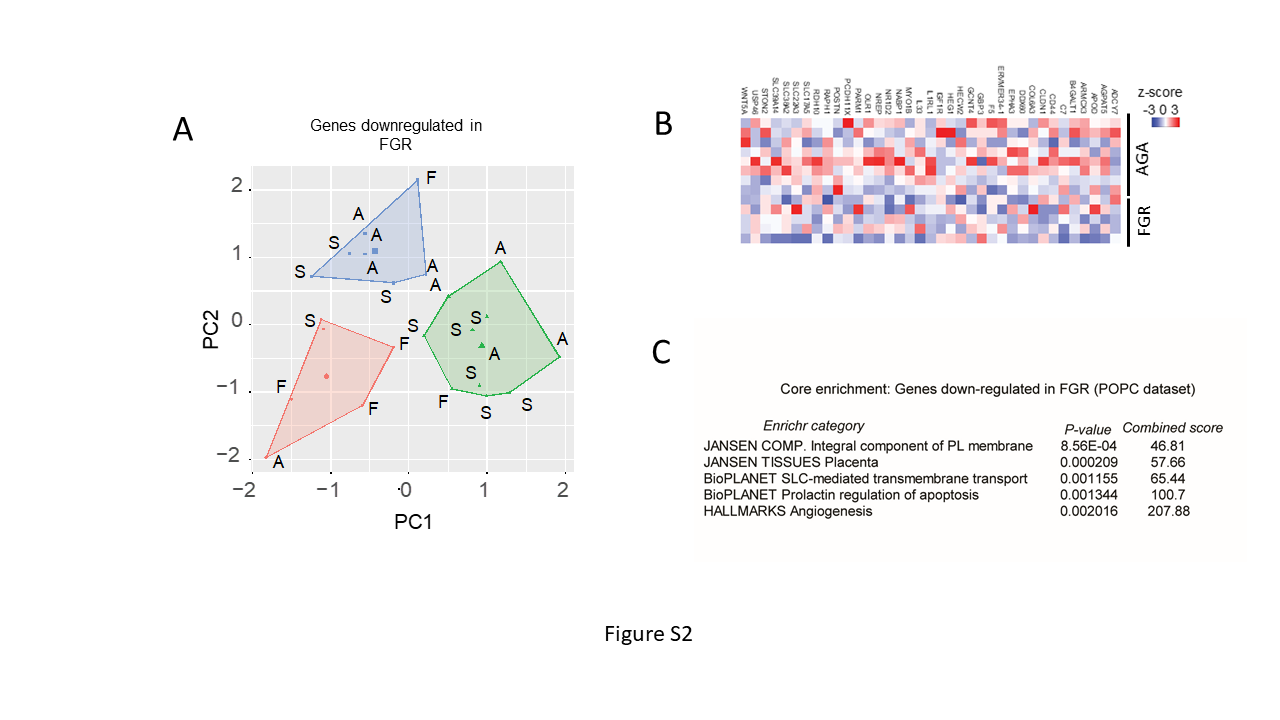


**Figure S4. A.** PCA of the indicated samples based on the signature reported in Table S4 (40 genes up-regulated in FGR). F=FGR, S=SGA, A=AGA. **B.** Heatmap of the expression levels (log2(Fc)) of the indicated genes in AGA, SGA and FGR samples in respect to the mean levels detected in AGA samples. **C.** Functional enrichment of the 40 gene signature obtained with Enrichr (<https://maayanlab.cloud/Enrichr/>).


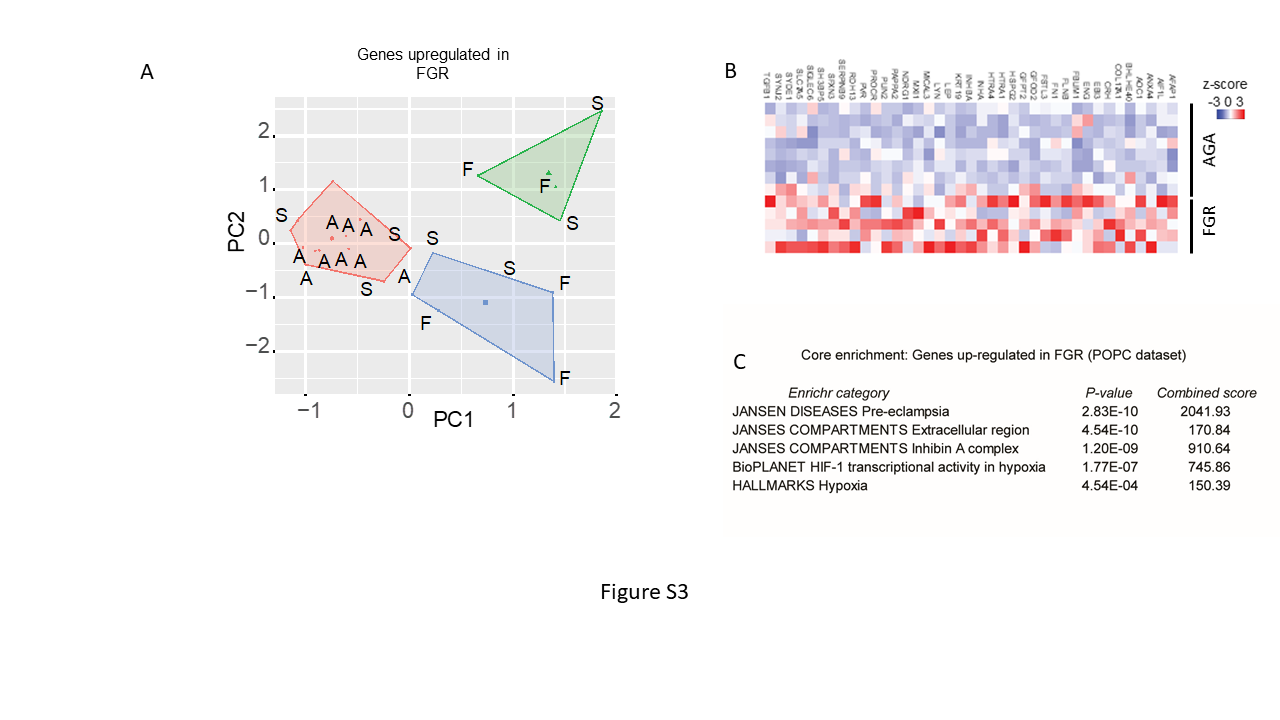


**Figure S5.** Heatmap of the expression levels (log2Fc) of the indicated genes in AGA, SGA and FGR samples in respect to the mean levels detected in AGA samples. Functional enrichment of the 65 gene signature (Table S5) obtained with Enrichr (<https://maayanlab.cloud/Enrichr/>).


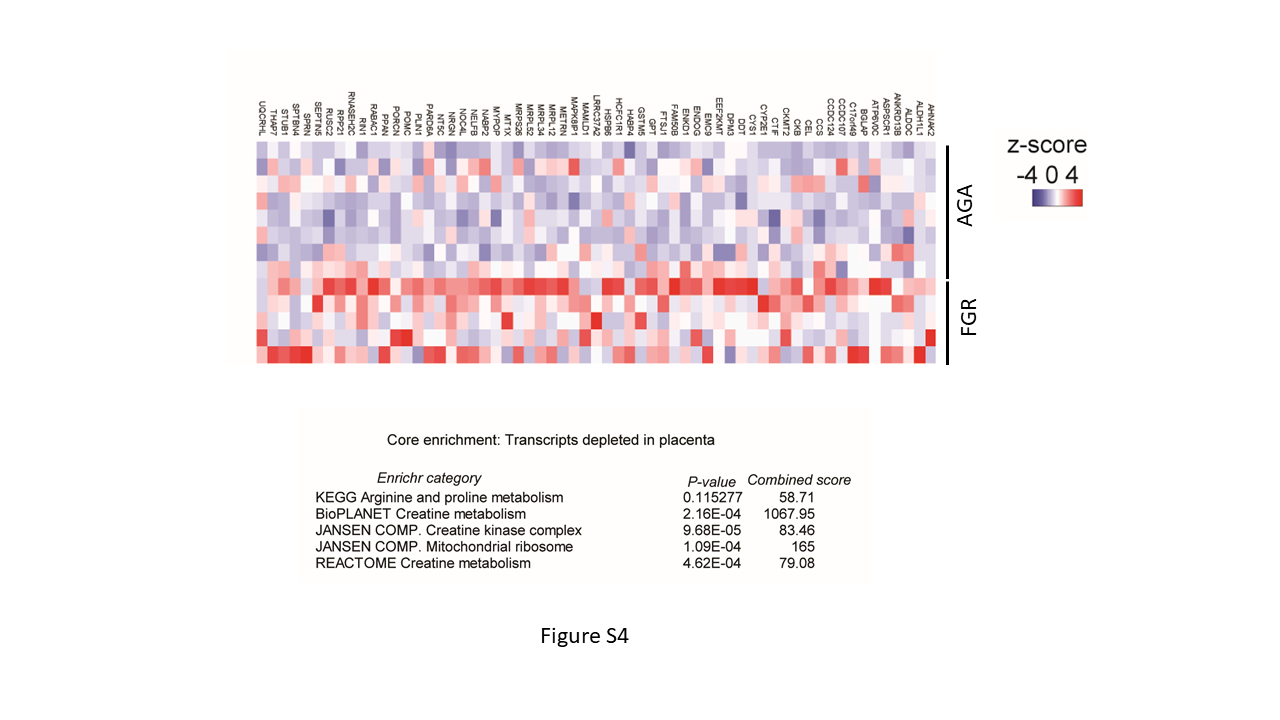


**Figure S6.** An unbiased metabolomic analysis performed on three FGR and three AGA placental biopsies allowed us to quantify 1,165 metabolites of which 19.4 % of the these metabolites exhibited differential abundance between AGA and FGR groups. PCA showed a clear separation between the AGA and FGR groups.


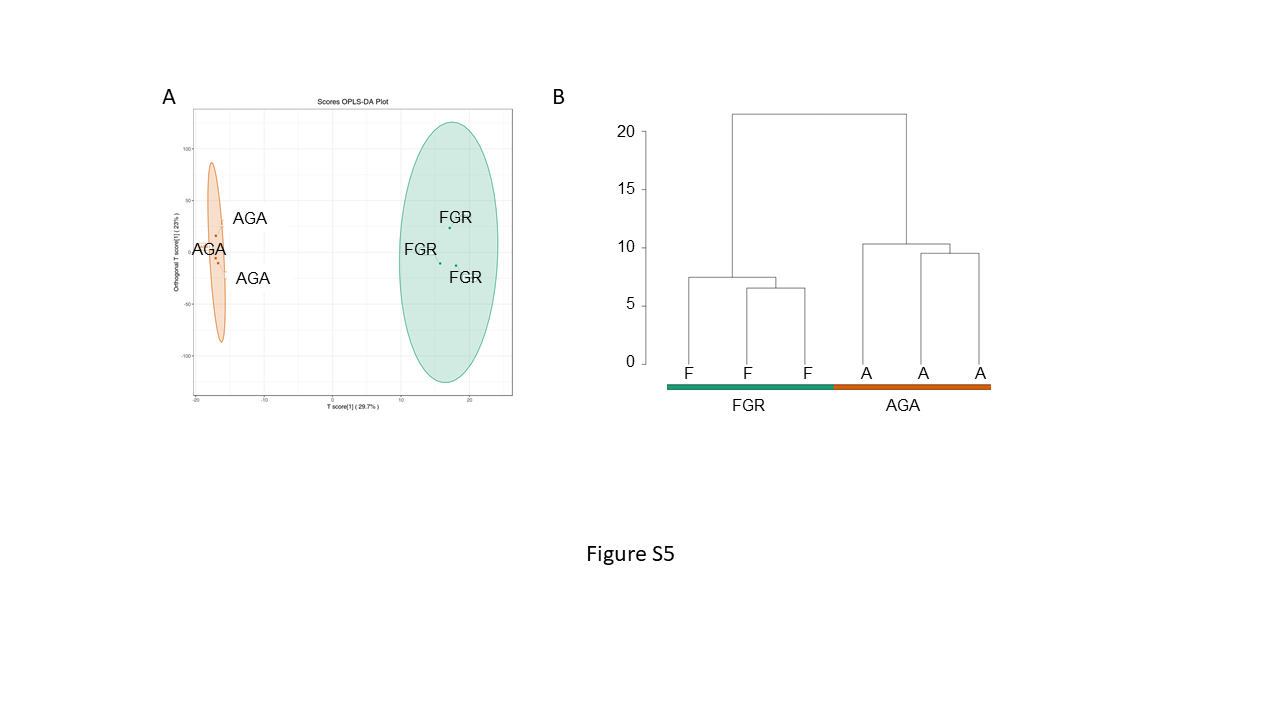


**Figure S7**. Heatmaps of DEGs associated with polyamine metabolism across AGA, SGA and FGR placentas in both male and female newborns. There is a clear difference in gene expression between AGA and FGR, especially for the SAT1 gene, which is upregulated in the FGR placentas regardless of the sex of the newborn. In contrast, SMS is only upregulated in placentas of male newborns.


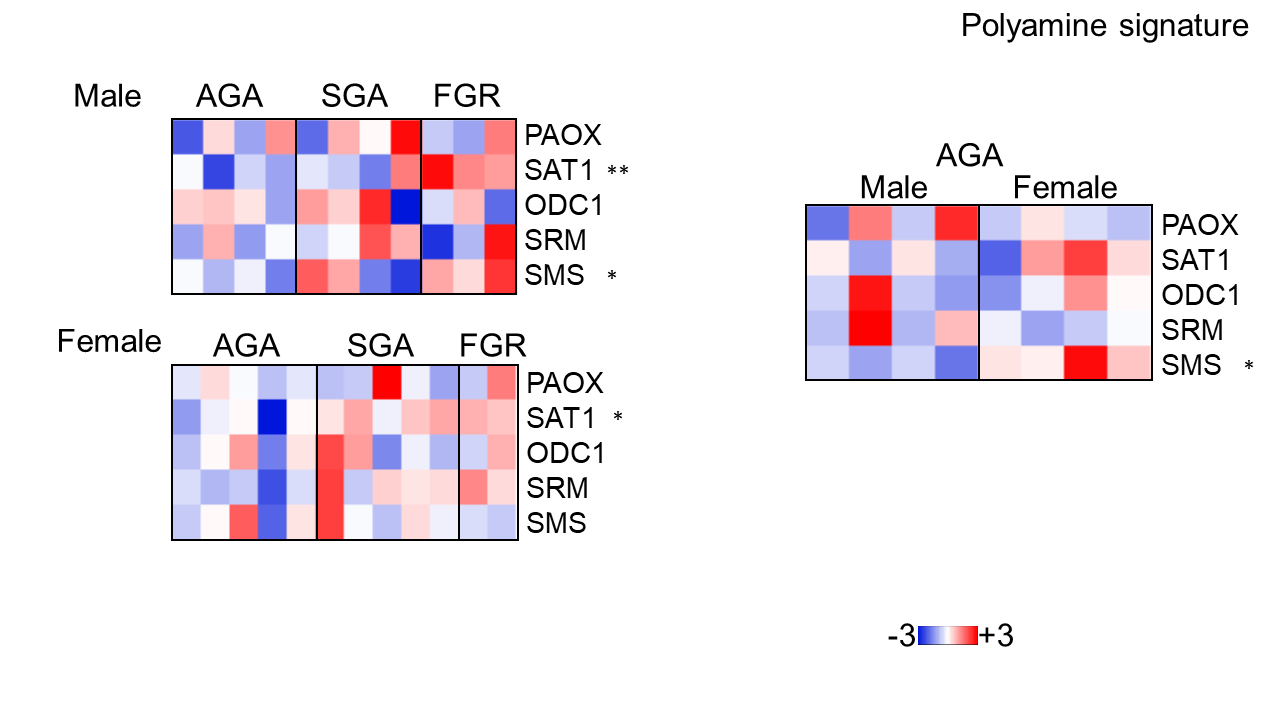

Supplement: Supplementary file 1 — Data S1. [file FSB2-38-e70222-s001.docx]
